# Importance of serological cross-reactivity among *Toxoplasma gondii, Hammondia* spp., *Neospora* spp., *Sarcocystis* spp. and *Besnoitia besnoiti*

**DOI:** 10.1017/S0031182017000063

**Published:** 2017-02-28

**Authors:** LUÍS F. P. GONDIM, JOSÉ R. MINEO, GEREON SCHARES

**Affiliations:** 1 Universidade Federal da Bahia, Escola de Medicina Veterinária e Zootecnia, Departamento de Anatomia, Patologia e Clínicas, Av. Adhemar de Barros, 500, Ondina, 40170-110 Salvador, Bahia, Brazil; 2 Laboratory of Immunoparasitology, Institute of Biomedical Sciences, Federal University of Uberlândia, Uberlândia, Brazil; 3 Friedrich-Loeffler-Institut, Federal Research Institute for Animal Health, Institute of Epidemiology, Südufer 10,17493 Greifswald-Insel Riems, Germany

**Keywords:** Serology, cross-reaction, *Toxoplasma gondii*, *Neospora* sp., *Sarcocystis* sp., *Hammondia* sp., *Besnoitia besnoiti*

## Abstract

*Toxoplasma gondii, Neospora* spp., *Sarcocystis* spp., *Hammondia* spp. and *Besnoitia besnoiti* are genetically related cyst-forming coccidia. Serology is frequently used for the identification of *T. gondii, Neospora* spp. and *B. besnoiti*-exposed individuals. Serologic cross-reactions occur in different tests among animals infected with *T. gondii* and *H. hammondi,* as well as among animals infected by *T. gondii* and *N. caninum*. Infections caused by *N. caninum* and *N. hughesi* are almost indistinguishable by serology. *Neospora caninum, B. besnoiti* and *Sarcocystis* spp. infections in cattle show some degree of serologic cross-reactivity. Antibody cross-reactivity between *Neospora* spp. and *H. heydorni*-infected animals is suspected, but not proven to occur. We review serologic cross-reactivity among animals and/or humans infected with *T. gondii, Neospora* spp., *Sarcocystis* spp., *Hammondia* spp. and *B. besnoiti*. Emphasis is laid upon antigens and serological methods for *N. caninum* diagnosis which were tested for cross-reactivity with related protozoa. Species-specific antigens, as well as stage-specific proteins have been identified in some of these parasites and have promising use for diagnosis and epidemiological surveys.

## INTRODUCTION

*Toxoplasma gondii, Neospora* spp., *Sarcocystis* spp., *Hammondia* spp. and *Besnoitia besnoiti* are closely related tissue cyst-forming parasites that belong to the family Sarcocystidae (Carreno *et al.* 1998; Mugridge *et al.* 2000). The complete life cycles of Sarcocystidae organisms are complex and involve several parasite stages in definitive and intermediate hosts. In species with known life cycles, carnivore or omnivore definitive hosts harbour sexual reproduction of the parasites in intestinal epithelium. They may shed large numbers of parasite oocysts or sporocysts in their feces. Intermediate hosts acquire infection upon ingestion of sporulated oocysts or sporocysts in food or water. Sporozoites invade intestinal epithelial cells of the intermediate hosts and spread to other tissues as tachyzoites or merozoites. These latter stages may encyst as slow multiplying forms, called bradyzoites. Ingestion of tissue cysts by carnivorism is the main route of infection for definitive hosts, which culminates with the formation of oocysts in their intestinal epithelium (Levine and Ivens, 1981).

Among the cyst-forming parasites, *T. gondii* is the most studied. It induces disease in a wide range of warm-blooded animals, including humans (Tenter *et al.* 2000), and causes abortion in livestock, especially in sheep and goats (Buxton, 1998). *Neospora caninum* was originally described in dogs (Bjerkas *et al.* 1984; Dubey *et al.* 1988*a*), but it gradually gained more attention from the scientific community as a major cause of neonatal mortality and abortion in mainly cattle but also other ruminants (O'Toole and Jeffrey, 1987; Parish *et al.* 1987; Anderson *et al.* 1991), besides causing neuromuscular disease in dogs (Ruehlmann *et al.* 1995). *Neospora hughesi* was proposed as a new species in the genus *Neospora* and has been associated with myeloencephalitis in horses (Marsh *et al.* 1998). The genus *Sarcocystis* possesses more than 100 species, with cattle as intermediate hosts of at least three species (*S. cruzi, S. hirsuta* and *S. hominis*). Among these, only *S. cruzi* is mildly pathogenic for cattle and generally non-pathogenic for its definitive host (dog) (reviewed by Dubey and Lindsay, 2006). Additional *Sarcocystis* spp. have been observed in bovine tissues, but their nomenclatures are still in debate (Dubey *et al*. 2016; Gjerde, 2016). Three parasite species compose the genus *Hammondia* (*H. hammondi, H. heydorni* and *H. triffittae*), which have no known association with disease in humans or in naturally infected animals. However, *Hammondia* spp. are closely related to *T. gondii* and *N. caninum* (Mugridge *et al.* 2000), so diagnostic methods need to discriminate between infections caused by these parasites. *Besnoitia besnoiti* causes a debilitating disease mainly characterized by both a cutaneous and systemic manifestation (Alvarez-Garcia *et al.* 2013). Reproductive abnormalities in cattle may also occur, such as infertility in bulls and abortion when cows are infected during pregnancy (Cortes *et al.* 2014). Bovine besnoitiosis was reported first more than a century ago in Southern France and Portugal, but the parasite has spread to several European countries during the last 10 years and besnoitiosis is now considered as a re-emerging disease in cattle, at least in Europe (Alvarez-Garcia *et al.* 2013).

*Toxoplasma gondii* and *H. hammondi* have cats as definitive hosts, which shed morphologically indistinguishable oocysts in their feces. Dogs and certain canid species serve as definitive hosts for *N. caninum* (McAllister *et al.* 1998; Gondim *et al.* 2004; King *et al.* 2010; Dubey *et al.* 2011) and *H. heydorni* (Blagburn *et al.* 1988; Slapeta *et al.* 2002; Soares *et al.* 2009). *Hammondia triffittae* has two species of wild canids (red fox and arctic fox) as definitive hosts (Gjerde and Dahlgren, 2011). Three *Sarcocystis* spp. from cattle, *S. cruzi, S. hirsuta* and *S. hominis*, have dogs, cats, and primates as definitive hosts, respectively (reviewed by Gjerde, 2016). *Besnoitia besnoiti* is suspected to have a carnivore as definitive host, but so far no animal has been identified shedding oocysts of the parasite by natural or experimental infections (Basso *et al.* 2011).

Infections caused by *T. gondii, Hammondia* spp., *Neospora* spp., *Sarcocystis* spp. and *B. besnoiti* are assessed by a great variety of diagnostic tools, depending on the purpose of the analysis and available biological sample. In clinically affected individuals, detection of parasite-specific antibodies in serum or other body fluids is the most commonly employed diagnostic approach, except for *Hammondia* spp., and *Sarcocystis* spp. from cattle. Continuous cultivation of bovine *Sarcocystis* spp., like *S. cruzi*, is difficult (Andrews *et al.* 1990). To date, *Hammondia* spp. cannot be continuously grown in cell culture, which impedes production of parasite antigens needed to produce serologic tests for these parasites (Riahi *et al.* 1995; Schares *et al.* 2003; Gondim *et al.* 2015). In this review, serologic cross-reactivity is reviewed in detail among infections caused by *T. gondii, Hammondia* spp., *Neospora* spp., *Sarcocystis* spp. and *B. besnoiti*. Special emphasis is put on serologic cross-reactivity among animals infected with *N. caninum* and related pathogens. Further consideration is given to the discovery and production of species-specific and stage-specific antigens, which promise to improve diagnostic specificity and may enable discrimination between different modes of parasite acquisition.

## SEROLOGY FOR *T. GONDII* AND CROSS-REACTIVITY WITH RELATED PATHOGENS

### Toxoplasma gondii *vs* H. hammondi

During the first decades after *T. gondii* was discovered, several scientists have attempted to develop serologic tests with high sensitivity and specificity to diagnose *T. gondii* infection, as well as to understand the antigenic composition of the parasite. The development and improvement of serological tests, such as the Sabin–Feldman dye test (DT) (Sabin and Feldman, 1948; Beverley and Beattie, 1952), direct agglutination test (Fulton and Turk, 1959; Desmonts and Remington, 1980), complement fixation test (CFT) (Sabin, 1949), enzyme-linked immunosorbent assay (ELISA) (Walls *et al.* 1977), immunofluorescence antibody test (IFAT) (Kelen *et al.* 1962), indirect haemagglutination test (IHA) (Lunde and Jacobs, 1958) and Western blot (WB) (Araujo *et al.* 1984) favoured a great advance in the study of toxoplasmosis.

#### Cross-immunity studies between *T. gondii* and *H. hammondi*

Mice and hamsters that were experimentally infected with *H. hammondi* (CR-4 strain) oocysts developed immunity and did not die after challenged with lethal doses of oocysts from a mouse-virulent *T. gondii* strain (M-7741 strain) (Frenkel and Dubey, 1975). In contrast, cats that were experimentally infected with *H. hammondi* and shed oocysts were not immunized against excretion of *T. gondii* oocysts (Frenkel and Dubey, 1975). An additional study approached cross-immunity between *T. gondii* and *H. hammondi*, by using six *H. hammondi* strains in an infection model of mice and hamsters (Christie and Dubey, 1977). The authors observed that 103 of 108 mice that were orally inoculated with *H. hammondi* oocysts survived a lethal challenge dose (10^5^ oocysts) of *T. gondii* (strain M-7741). The *H. hammondi*-inoculated hamsters also developed immunity against a lethal dose of *T. gondii* oocysts, but this immunity was variable depending on the *H. hammondi* strain. The two most immunogenic *H. hammondi* strains conferred protection to fatal toxoplasmosis in 100 and 83% of the hamsters, respectively, after challenging with *T. gondii* oocysts (Christie and Dubey, 1977).

Immunization of goats with *H. hammondi* had a protective effect against abortion induced by *T. gondii*; however, the immunization did not prevent transplacental transmission of *T. gondii* in pregnant does (Munday and Dubey, 1988). Partial immunity against toxoplasmosis was also obtained in Tamar wallabies (*Macropus eugenii*), that were orally infected with 1 × 10^5^ oocysts of *H. hammondi* (Reddacliff *et al.* 1993).

#### Serologic cross-reactivity between *T. gondii* and *H. hammondi*

When *H. hammondi* was first described, some rodent species experimentally infected with this parasite developed cross-reacting antibodies against *T. gondii* antigens in the DT (Frenkel and Dubey, 1975). Further studies were carried out and confirmed that serologic cross-reactivity between *T. gondii* and *H. hammondi* occurred with sera from other animals, besides rodents. Weiland *et al.* (1979) investigated cross-reactivity between *T. gondii* and *H. hammondi* in four animal species (120 mice, six dogs, six rabbits and six pigs) by using five serological tests (DT, CFT, ELISA, IFAT and IHA). Half of the animals were orally inoculated with *T. gondii* oocysts and the other half received *H. hammondi* oocysts by the same route. Sera from *H. hammondi*-infected mice reacted with *T. gondii* antigens in three tests (DT, ELISA and CFT). Sera from dogs infected with *H. hammondi* recognized *T. gondii* antigens by DT and ELISA. Sera from rabbits exhibited cross-reaction between the two parasites by ELISA. The sera of pigs infected with *H. hammondi* did not cross-react with *T. gondii* in any of the five serological tests. In this study, the IFAT was considered the most *Toxoplasma*-specific method. During the course of infection, the animals infected with *T. gondii* presented higher titres in the tests when compared with those infected with *H. hammondi* (Weiland *et al.* 1979). Munday and Dubey (1986) observed that sheep that were inoculated with *H. hammondi* oocysts presented cross-reactivity with *T. gondii* antigen by IFAT. Before infection with *H. hammondi*, the sheep had no detectable antibodies to *T. gondii*. After oral inoculation with *H. hammondi* oocysts, the animals presented antibody titres of 1:16 to *T. gondii* by IFAT. Goats infected with *H. hammondi* were shown to produce antibodies against *T. gondii* tested by DT, with titres up to 1:64 (Dubey, 1981).

The antigenic similarity between *T. gondii* and *H. hammondi* was investigated using sera from experimentally infected mice (Araujo *et al.* 1984). The authors employed *T. gondii* tachyzoites (RH strain) for two antigen detection procedures: (1) the antigen was labelled with ^125^I, immune-precipitated with sera from *T. gondii* or *H. hammondi*-infected mice, run by SDS–polyacrylamide gel electrophoresis (SDS–PAGE) and autoradiographed; (2) the antigen was lysed, separated by SDS–PAGE and tested by immunoblot. The sera from mice that were orally inoculated with *H. hammondi* oocysts recognized *T. gondii* antigens of MW 92·5 kDa (two antigens), 66·2 kDa, between 66·2 and 45 kDa, and between 31 and 21·5 kDa; compared with the *T. gondii*-positive reaction only a *T. gondii* antigen of 21·5 kDa was not recognized by the *H. hammondi*-positive mouse serum (Araujo *et al.* 1984). This study confirmed that *T. gondii* and *H. hammondi* have similar antigenic components in their tachyzoites.

To facilitate scientific communication among research groups and laboratories, Sibley *et al.* (1991) proposed a nomenclature system for naming mutants, genes and gene products of *T. gondii*, which was based on the system used for the yeast *Saccharomyces cerevisiae.* As an example, the surface protein (P30) was designated SAG1, which is the product of the *SAG1* gene. However, as it was observed more recently that *SAG1* genes belong to a superfamily of related genes, named *SRS* (SAG1-related sequences), which encode a superfamily of structurally related surface proteins from *T. gondii*, the name of SAG1 (P30) has been changed to SRS29B (Wasmuth *et al.* 2012).

Riahi *et al.* (1998) studied the antigenic similarity of *T. gondii* and *H. hammondi* employing five monoclonal antibodies (MAbs) against *T. gondii* surface antigens and a polyclonal mouse serum to *H. hammondi*. In order to produce enough *H. hammondi* antigen, the authors used an *in vitro* model that allows the production of *H. hammondi* cysts up to 3 months in cell culture (Riahi *et al.* 1995). The cyst formation, confirmed by ultrastructural characteristics of the organism, started from 6 days after sporozoites were inoculated into feline kidney cells (CRFK). At 4 days of infection, the authors assumed that the multiplying *H. hammondi* zoites were tachyzoites (Riahi *et al.* 1998).

Tachyzoite antigens from *T. gondii* and *H. hammondi* were tested by IFAT and WB. By combining the two serologic techniques (IFAT and WB), five *T. gondii* antigens (MW of 30, 32, 35, 66 and 90 kDa) were recognized using polyclonal anti-*H. hammondi* serum. An interesting finding obtained by Riahi *et al.* (1998) was the recognition of the SAG1 (SRS29B) antigen by the anti-*H. hammondi* serum, as this protein is a major antigen of *T. gondii* (Burg *et al.* 1988) and had been considered to be a specific marker for the parasite (Mineo *et al.* 1993). Despite the antigenic similarity between *T. gondii* and *H. hammondi*, MAbs targeted against *H. hammondi* antigens were produced, and five of them did not cross-react with *T. gondii* antigens (Riahi *et al.* 2000). These findings are promising for the characterization of *H. hammondi*-specific antigens or epitopes, which could enable development of serologic tests for this parasite that would not cross-react with *T. gondii*.

The genome of a German strain of *H. hammondi* was sequenced and the genomic synteny between this parasite and *T. gondii* was higher than 95% (Walzer *et al.* 2013). It was found that orthologues of key *T. gondii* mouse virulence genes are functionally conserved in *H. hammondi*, but these data were not enough to explain the phenotypic differences observed between both parasites (Walzer *et al.* 2013). In a recent work, the genomes of 62 strains of *T. gondii* were compared with those from *H. hammondi, N. caninum* and *Sarcocystis neurona* (Lorenzi *et al.* 2016); these authors demonstrated that *T. gondii* possesses an expansion of parasite-specific secretory pathogenesis determinants (SPDs) when compared with the three latter parasites. The SPDs encompass genes encoding secretory proteins from micronemes, dense granules, rhoptries and surface antigens, whose expansion and diversity are associated with the patterns of transmission, host range and pathogenicity of *T. gondii* (Lorenzi *et al.* 2016). It was reported that *T. gondii* shares a high number of orthologues with *H. hammondi* (7095) and *N. caninum* (6308) (Lorenzi *et al.* 2016).

The search for more practical and efficient methods to detect specific antibodies against *T. gondii* pushed the establishment of tests based on antigenic fractions, which are gradually replacing traditional tests based on whole organisms (agglutination, DT and IFAT) or total extracts of parasite antigens. Recognized immunodominant antigens of *T. gondii*, including the tachyzoite surface antigens SAG1 (p30) (Kasper, 1987; Mineo *et al.* 1993), SAG2 (p22) (Prince *et al.* 1990), SAG3 (P43) (Cesbron-Delauw *et al.* 1994), SAG4 (p18) (Odberg-Ferragut *et al.* 1996) and several other proteins from dense granules, rhoptries and micronemes are being produced as recombinant proteins (reviewed by Holec-Gasior, 2013).

So far, it is not known whether humans may be infected with *H. hammondi*, and in case it happens, the possibility of serologic cross-reactivity with *T. gondii* antigens cannot be ruled out. Cats seem to shed *T. gondii* and *H. hammondi* in similar proportions as reported in a recent study from Germany (Schares *et al*. 2016), and humans and animals of many species are potentially exposed to *H. hammondi*. The establishment of *H. hammondi* infections in most animal species has not been rigorously investigated; however, the life cycle of *H. hammondi* seems to lack avian hosts (Dubey and Sreekumar, 2003).

### Toxoplasma gondii *vs* N. caninum

The serologic differentiation between *T. gondii* and *H. hammondi* infections has clinical and epidemiological relevance. Since *H. hammondi* is not known to induce disease in animals or humans, the major concern about the serologic differentiation of these parasites seems to be to avoid *T. gondii* false-positive results in individuals potentially infected with *H. hammondi*. The identification of *N. caninum* (Bjerkas *et al.* 1984; Dubey *et al.* 1988*a*) imposed a new challenge for the scientific community, because this non-zoonotic Toxoplasmatinae parasite is able to infect and cause disease in some mammalian animals that are also susceptible to *T. gondii* infection (Buxton, 1998). The comparison of the whole-genome sequences from *T. gondii, H. hammondi* and *N. caninum* showed that these three related tissue-cyst-forming coccidian parasites have a similar total genome size of 62–65 Mb, and many similar orthologous groups of proteins involved in key biological functions (Lorenzi *et al.* 2016).

*Neospora caninum* can be maintained as tachyzoites in cell culture (Dubey *et al.* 1988*b*), which has enabled the development of a range of serological tests and antibodies against the parasite (Björkman and Uggla, 1999). Neosporosis research was accelerated by the prior accumulation of knowledge about and procedures for *T. gondii*.

The nomenclature system adopted for mutants, genes and gene products of *T. gondii* (Sibley *et al.* 1991) was proposed for *N. caninum* (Howe and Sibley, 1999). In the comparison of *T. gondii* and *N. caninum* homologous antigens (e.g. SAG1), the use of a Tg or Nc prefix was recommended to distinguish these gene products (e.g. TgSAG1 and NcSAG1) (Howe and Sibley, 1999). Also, a cluster of cell-surface genes was found in *N. caninum*, as described for *T. gondii*, and these gene products were re-named as members of the SRS superfamily (Wasmuth *et al.* 2012).

#### Cross-immunity studies between *T. gondii* and *N. caninum*

Cross-protection of mice immunized with *N. caninum* and challenged with *T. gondii* is probably *T. gondii* strain- and dose-dependent. In one study, mice were immunized with *N. caninum* and died after challenging with the highly virulent RH strain of *T. gondii* (Lindsay *et al.* 1990). When mice were immunized with *N. caninum* and challenged with a less-virulent *T. gondii* strain (PLK, a clone from the ME49 strain), the animals had 100% protection against death; immunization with a higher dose of *N. caninum* tachyzoites improved protection against *T. gondii*-induced disease (Kasper and Khan, 1998). Similar levels of protection were observed when mice were immunized with *N. caninum* tachyzoites and then challenged with oocysts of *T. gondii* from a moderately virulent strain (Lindsay *et al.* 1998). In another study, pregnant sheep were immunized with a *T. gondii* sheep vaccine (Toxovax^®^, Intervet, Cambridge, UK) and challenged with a high dose of *N. caninum* tachyzoites (10^7^ tachyzoites per animal) at 90 days of gestation (Innes *et al*. 2001). No protection against fetal death was observed. The authors speculated that if the sheep were challenged with a lower dose of *N. caninum* tachyzoites, there perhaps would be some degree of cross-immunity (Innes *et al*. 2001). The confirmation of cross-immunity between *T. gondii* and *N. caninum* in some studies suggests that these parasites share antigens which may also be involved in serologic cross-reactivity.

#### Serologic cross-reactivity between *T. gondii* and *N. caninum*

In the initial observation of *N. caninum* in dogs, sera from five animals that were naturally infected with the parasite tested negative for *T. gondii* by the DT (Bjerkas *et al.* 1984). In addition, dogs that were naturally or experimentally infected with *N. caninum* did not cross-react to *T. gondii* by IFAT, when using 1:50 dilutions as cutoff (Dubey *et al.* 1988*b*). Accordingly, the same or higher dilution cutoffs by IFAT have been found to be appropriate to avoid cross-reactivity between *N. caninum* and *T. gondii* in serum samples from different hosts (Lobato *et al.* 2006; Silva *et al.* 2007; Benetti *et al.* 2009). Apical reactions, i.e. reactions limited to the apex of the parasite were regarded as non-specific in *N. caninum* IFAT. Cross-reactivity with apical antigens is potentially caused by the high conservation of antigens in the apical organelles of a variety of Apicomplexan parasites, including *T. gondii*. In contrast, a complete peripheral fluorescence of the parasite was considered as a positive response (Pare *et al.* 1995*b*).

Cross-reactive antigens between *N. caninum* and *T. gondii* have been observed by immunohistochemistry using tissues of naturally- or experimentally infected animals. A rabbit anti-*N. caninum* serum cross-reacted with *T. gondii* in tissue sections from mice (Barr *et al.* 1991). A bradyzoite antigen from *T. gondii*, designated as BAG1 (synonymous to BAG5) (Weiss *et al.* 1992; Parmley *et al.* 1995), was used to produce hyperimmune serum in rabbit, which cross-reacted with bradyzoites of *N. caninum* (McAllister *et al.* 1996). Polyclonal sera against *T. gondii* tachyzoites induced strong cross-reactivity with *N. caninum* tachyzoites by immunohistochemistry (Sundermann *et al.* 1997). The use of MAbs specific to *T. gondii* (Sundermann *et al.* 1997) or a combination of two MAbs specific to *N. caninum* (Uzêda *et al.* 2013), were demonstrated to avoid immunohistological cross-reactivity between these protozoa.

Although species-specific MAbs have been developed, serologic cross-reactions between *T. gondii* and *N. caninum* have been shown to occur also by means of MAbs. Sundermann *et al.* (1997) generated MAbs against *T. gondii* tachyzoites and observed among 26 MAbs tested by IFAT, five antibodies that cross-reacted with *N. caninum* tachyzoites. Kobayashi *et al.* (2013) cloned the *NcBAG1* gene and generated MAbs against its recombinant protein, which recognized TgBAG1. Liao *et al.* (2005*a*) produced 384 MAbs against *N. caninum* by immunizing mice with *N. caninum* tachyzoites; 10 of the 384 MAbs were also reactive against *T. gondii* tachyzoites. Similarly, Sohn *et al.* (2011) developed 46 MAbs using a mouse immunized with a mixed fraction of *N. caninum* organelles and some of the MAbs cross-reacted with *T. gondii*. MAbs generated to oocyst antigens of *T. gondii* cross-reacted by immunofluorescence with the sporocyst wall (Dumetre and Darde, 2007) and tissue the cyst wall of *N. caninum* (Gondim *et al.* 2016). These findings show that cross-reactive antigens between *T. gondii* and *N. caninum* are present in tachyzoites, tissue cysts and oocysts of these parasites.

Affinity-purified antibodies raised against a 38 kDa microneme-associated protein of *N. caninum* (NcMIC3) also recognized a 45 kDa protein in tachyzoite extracts of *T. gondii* (Sonda *et al.* 2000). MAbs raised against *T. gondii* reacted with *N. caninum* tachyzoites by IFAT, but only at low titres (10–40) (Latif and Jakubek, 2008). A number of reports demonstrated the usefulness of MAbs against *T. gondii*, which do not cross-react with *N. caninum* (Baszler *et al.* 2001; Uchida *et al.* 2004; Srinivasan *et al.* 2006; Cunha-Junior *et al.* 2010).

Certain surface antigens of *N. caninum*, although not identical to those from *T. gondii*, were homologous to them (Hemphill *et al.* 1997; Howe *et al*. 1998; Howe and Sibley, 1999). Two surface antigens of *N. caninum*, similar to SAG1 and SRS2 from *T. gondii*, were called NcSAG1 and NcSRS2. MAbs against NcSAG1 (6C11, Ncmab-4) did not cross react with *T. gondii* (Björkman and Hemphill, 1998; Howe *et al*. 1998). Evaluation of several MAbs against NcSRS2 (5H5, Ncmab-10, 5·2·15) revealed no cross-reactions with *T. gondii* antigens in immunoblot (Björkman and Hemphill, 1998; Howe *et al*. 1998; Schares *et al.* 1999*a*).

Antigens of *N. caninum* tested by WB using monoclonal or polyclonal antibodies, resulted in the identification of a limited number of specific immunodominant bands (often referred to as ‘immunodominant antigens’) and other less reactive bands (Table 1). The comparison of these antigens based on their molecular weights is difficult, as differences on the SDS-PAGE conditions, especially the use of reducing or non-reducing conditions, cause variation in the estimated molecular weights. Most likely, some of these immunodominant bands observed in WBs may represent more than a single protein.

**Table 1.** Immunodominant bands recognized by *Neospora caninum*-infected or immunized animals in tachyzoite antigen

Antigen treatment	Molecular weight in kDa of antigenic bands (antigen treatment)^a^	Source of *N. caninum* antibodies	Parasite species used to examine specificity	Reference
Non-reduced	16, 29, 31, 32, 37, 46, 51, 56, 79 (SDS–PAGE sample buffer soluble total lysate)	Hyperimmune serum from a rabbit	Not examined	(Barta and Dubey, 1992)
	17, 29, 30, 37, 46, 88, 97 (aqueous buffer and detergent soluble total lysate)	Sera from several naturally and experimentally *N. caninum* infected (cattle, dog, sheep, goat, pig) or immunized animal species (rabbits)	*T. gondii, S, cruzi, H. hammondi*; faint reactions to 46 kDa observed in pre-immune serum of rabbits; 46, 88, 97 kDa antigens clearly and 30 kDa faintly and inconsistently recognized by *T. gondii* antiserum; no cross-reactivities reported with anti-*S. cruzi* or anti-*H. hammondi*	(Bjerkas *et al.* 1994)
	17–18, 30–45, >51 (soluble part of sonicated total lysate and iscom incorporated antigen)	Serum from experimentally infected or immunized rabbits and from a naturally infected dogs	*T. gondii, S. cruzi*; anti-*T. gondii* showed faint reactions with 40 kDa; anti-*S. cruzi*: no reactions	(Björkman *et al.* 1994)
	28–35, 45–52, 64–78 (ISCOM incorporated antigen)	Sera and milk from naturally and experimentally infected bovines	*T. gondii*; serum of a *T. gondii*-infected calf did not react	(Björkman *et al.* 1997)
	28, 31, 40, 83, 91 (soluble part of sonicated total lysate)		*T. gondii, S. cruzi*; 40 kDa faintly stained by anti-*T. gondii*; anti-*S. cruzi* showed negligible reactions	(Stenlund *et al.* 1997)
	16/17, 29/30, 30–36, 37, 45 (SDS–PAGE sample buffer soluble total lysate)	Sera from experimentally infected calves and naturally infected cattle	*T. gondii*; no cross-reactions with anti-*T. gondii*	(Atkinson *et al.* 2000)
	17, 29, 30, 33, 37, 40 (SDS–PAGE sample buffer soluble total lysate)	Sera from naturally infected bovine fetuses	Not examined	(Sondgen *et al.* 2001)
	29, 36 (SDS–PAGE sample buffer soluble total lysate)	Sera from naturally infected bovines	*T. gondii;* no cross-reactions in 14 of 17 naturally seropositive cattle, faint reactions in the remaining (true infection status regarding *N. caninum* unknown)	(Staubli *et al.* 2006)
	14, 29, 30, 33, 35, 40, 55, 60, 77, 84, 97, 170 (aqueous buffer and detergent soluble total lysate)	Sera from experimentally and naturally infected dogs	Not examined	(Jesus *et al.* 2007)
	17, 29–32 (aqueous buffer soluble total lysate)	Sera from naturally infected dogs and mice	*T. gondii*; cross-reactivity with 45–50, 60, 82, 88, 97 and 120 kDa antigens as determined by immunoblot inhibition assays	(Silva *et al.* 2007)
Reduced	17, 31, 34, 37, 40·5, 47, 50, 55, 65 (SDS–PAGE sample buffer soluble total lysate)	Hyperimmune serum from a rabbit	Not examined	(Barta and Dubey, 1992)
	14, 20, 31, 35, 42, 54, 65, 76, 116 (aqueous buffer soluble total lysate)	Sera of cattle naturally or experimentally infected with *N. caninum, T. gondii, S. cruzi, S. hirsuta, S. hominis*	*T. gondii, S. hirsuta*: 14, 31, 37, 42, 55, 80 kDa recognized by anti-*T. gondii*; 11, 25, 34, 37 kDa recognized by anti-*S. hirsuta* serum.	(Baszler *et al.* 1996)
	36·5–38, 45·5–48·5, 52–53·5, 58, 58·5, 59·5, 60·5, 62, 63·5, 64, 66·5, 67, *67·5*, 68·5, 69·5 (total lysate antigen)	*N. caninum* or *T. gondii* experimentally infected cattle, sheep and goats	*T. gondii*; 63·5, 64, 66·5, 67, 67·5, 68·5, 69·5 kDa antigens recognized	(Harkins *et al.* 1998)
	17–18, 34–35, 37, 60–62 (aqueous buffer soluble total lysate)	Sera from naturally infected pregnant cattle and aborted fetuses	Not examined	(Alvarez-Garcia *et al.* 2002)
	18, 25, 33, 35–36, 45–46, 47, 60–62 (SDS–PAGE sample buffer soluble total lysate)	Sera from naturally infected bovines	Not examined	(Okeoma *et al.* 2004)

a Cross-reacting immunodominant antigen bands underlined.

In WB, non-reduced antigens exhibit much stronger reactivity than reduced antigens (Barta and Dubey, 1992) which is a clear indication that most of the epitopes recognized on these antigens are conformational epitopes. Under non-reduced conditions a large number of researchers observed mainly four areas with specific immunodominant bands in *N. caninum* tachyzoite antigen: 14–19 kDa, 29–32 kDa, 30–36 kDa, and 36–40 kDA. However, other areas with major bands of reactions were also observed with non-reduced antigens, but differed widely between studies (Table 1).

Sera from animals immunized with recombinant forms of the major *N. caninum* antigens NcSAG1 and NcSRS2 provided evidence that these antigens are among those recognized in the range 29–32 kDa and 36–40 kDA bands (Howe *et al*. 1998) (Table 2).

**Table 2.** *Neospora caninum*-recombinant antigens and cross-reactions tested against *Toxoplasma gondii* and related protozoan parasites

*N. caninum*-recombinant antigens established for diagnostic purposes (other names or variants of the recombinant proteins)	Localisation (estimated size, reference)	Parasite species against which cross-reactivity was tested	References which examined cross-reactivity
rNcSAG1 (rNcp29, NcSAG1t)	Surface (29–38 kDa) (Howe *et al.* 1998; Sonda *et al.* 1998)	*T. gondii*	(Howe *et al.* 2002; Chahan *et al.* 2003; Liao *et al.* 2005*b*; Huang *et al.* 2007; Ybanez *et al.* 2013)
		*Sarcocystis* spp.	(Howe *et al.* 2002)
rNcSRS2 (Ba/SRS2p44, NcSRS2t, tNcSRS2)	Surface (35–43 kDa) (Hemphill and Gottstein, 1996; Hemphill *et al.* 1997; Howe *et al.* 1998)	*T. gondii*	(Nishikawa *et al.* 2001, 2002; Gaturaga *et al.* 2005; Huang *et al.* 2007; Liu *et al.* 2007)
		*H. heydorni*	(Nishikawa *et al.* 2001, 2002)
rNcSAG4	Surface (18 kDa) (Fernandez-Garcia *et al.* 2006)	*T. gondii*	(Aguado-Martinez *et al.* 2008)
		*B. besnoiti*	(Aguado-Martinez *et al.* 2008)
		*Sarcocystis* spp.	(Aguado-Martinez *et al.* 2008)
rNcGRA6 (Nc14·1, NcDG2, NcGRA6d, NcGRA6s)	Dense granules (68 kDa, 37 kDa) (Liddell *et al.* 1998)	*T. gondii*	(Lally *et al.* 1996; Jenkins *et al.* 1997)
		*Sarcocystis* spp.	(Lally *et al.* 1996; Jenkins *et al.* 1997)
rNcGRA7 (Nc4·1, NcDG1, N57, MBPNcGRA7, rNcGRA7t, rNcGRA7)	Dense granules (33 kDa, 17–18 kDa) (Lally *et al.* 1997; Hemphill *et al.* 1998; (Alvarez-Garcia *et al.* 2007; Fuchs *et al.* 1998)	*T. gondii*	(Lally *et al.* 1996; Jenkins *et al.* 1997; Aguado-Martinez *et al.* 2008; Hamidinejat *et al.* 2015)
		*B. besnoiti*	(Aguado-Martinez *et al.* 2008)
		*Sarcocystis.* spp.	(Lally *et al.* 1996; Jenkins *et al.* 1997; Aguado-Martinez *et al.* 2008)
		Others, including *Babesia* spp., *Tritrichomonas fetus, Leishmania infantum*	(Louie *et al.* 1997; Hara *et al.* 2006; Huang *et al.* 2007)
rNcMIC10 (NcMiC10 M, NMIC10N, NcMIC10C)	Micronemes (18 kDa) (Yin *et al.* 2012)	*T. gondii*	(Yin *et al.* 2012)
rSubtilisin-like serin protease (N54, NcSUB1, NcSUB1t, NcSUB1tr)	Unknown (97, 87, 77, 67 and 64 kDa) (Louie *et al.* 1997)	*T. fetus*	(Louie *et al.* 1997)

Under reduced conditions there is a dominant band between 17–19 kDa which most likely represent reactions against NcGRA7 (Alvarez-Garcia *et al.* 2007) and other antigens (Table 2). However, as demonstrated by MAbs (4·7·12; Ncmab-7) in combination with immunoprecipitation, surface biotinylation and immuno-electron microscopy, a surface antigen might also be among those migrating in WB at 17–19 kDa under reduced conditions (Björkman and Hemphill, 1998; Schares *et al.* 1999*a*). Further immunodominant banding areas are at 34–36 and 37–46 kDa, eventually also representing reactions to NcSAG1, NcGRA6, NcGRA7 and NcSRS2 (Table 2). When tested with polyclonal antibodies against *T. gondii*, only minor reactions were observed with antigen bands regarded as specific for *N. caninum* in WB (Barta and Dubey, 1992; Björkman *et al.* 1994, 1997).

For the diagnosis of *T. gondii* infection in veterinary investigations, the use of recombinant and synthetic antigens, developed using novel molecular techniques, have expanded diagnostic options as alternatives to native antigens directly isolated from cultivated parasites. For diagnosis of *T. gondii* infection in cats, sheep and pigs, some species-specific ELISAs are available that have performed well when compared with previous reference serological techniques, as reviewed by Wyrosdick and Schaefer (2015).

ELISAs for *N. caninum* antibodies in sera from dogs and cattle have been developed in several studies (Table 3). Conventional ELISAs using crude soluble antigen showed higher levels of serologic cross-reactivity to *T. gondii* when compared with IFAT (Björkman *et al.* 1994; Silva *et al.* 2007). Serologic cross-reactivity with *T. gondii* was also observed when polyclonal mouse and cat sera were tested by a *N. caninum* ELISA using crude antigen (Nishikawa *et al.* 2002). In contrast, the same mouse and cat sera did not cross-react by an ELISA based on *N. caninum* recombinant antigen (NcSRS2) (Nishikawa *et al.* 2002). ELISAs prepared with *N. caninum* tachyzoite antigen associated with immunostimulating complexes (ISCOM), and using MAbs as secondary antibodies, presented better specificity than conventional ELISAs (Björkman *et al.* 1994, 1997). The ISCOM particles have affinity for surface proteins, which minimizes interference by internal non-specific antigens (Björkman *et al.* 1994). Moreover, none of the MAbs developed against *N. caninum* ISCOM incorporated antigens (including also Ncmab-4, and Ncmab-10 mentioned above) cross-reacted with *T. gondii* (Björkman and Lunden, 1998). An ELISA based on immuno-affinity-purified native NcSRS2 showed no significant cross-reactions when tested with sera from cattle experimentally infected with a variety of protozoan parasites including also ten cattle infected with *T. gondii* (Schares *et al.* 2000). In addition, the TgSAG2A molecule has been demonstrated to be specific to *T. gondii*, considering that no cross-reactivity has been shown with *N. caninum* when using recombinant protein or even mimotopes derived from this molecular marker, as characterized by A4D12 MAb (Bela *et al.* 2008; Carvalho *et al.* 2008; Cunha-Junior *et al.* 2010; Santana *et al.* 2012; Macedo *et al.* 2013).

**Table 3.** Cross-reactions tested for *Neospora caninum* in published in-house ELISAs

*N. caninum* ELISA	Parasitic species for which cross-reactivity was examined	Reference
Whole tachyzoite lysate antigen ELISA (various extraction protocols)	*T. gondii*	(Pare *et al.* 1995*a*; Dubey *et al.* 1996)
	*Hammondia* spp.	(Dubey *et al.* 1996)
	*Sarcocystis.* spp.	(Pare *et al.* 1995*a*; Dubey *et al.* 1996)^a^
	*Eimeria* spp.	(Pare *et al.* 1995*a*; Dubey *et al.* 1996)
	*Cryptosporidium* spp.	(Pare *et al.* 1995*a*; Dubey *et al.* 1996)
	*T. gondii, Sarcocystis* spp., *Babesia* spp.	(Osawa *et al.* 1998)
	*T. gondii, Sarcocystis* spp., *Eimeria* spp., *Babesia* spp., *Cryptosporidium* spp.	(Wouda *et al.* 1998)
	*T. gondii*	(Silva *et al.* 2007)
Fixed whole tachyzoites ELISA	*T. gondii*	(Williams *et al.* 1997)
	*Sarcocystis* spp.	(Williams *et al.* 1997)^a^
	*Eimeria* spp.	(Williams *et al.* 1997)
	*Babesia* spp.	(Williams *et al.* 1997)^a^
	*Cryptosporidium* spp.	(Williams *et al.* 1997)
ISCOM incorporated antigen, indirect	*T. gondii*	(Björkman *et al.* 1994)^b^, (Björkman *et al.* 1997), (Björkman and Hemphill, 1998)^c^, (Björkman and Lunden, 1998)^d^,
	*Sarcocystis* spp.	(Björkman *et al.* 1994)^b^, (Björkman *et al.* 1997)
	*Eimeria* spp., *Babesia* spp.	(Björkman *et al.* 1997)
Native NcSRS2, indirect	*T. gondii*	(Schares *et al.* 2000; Hosseininejad *et al.* 2010)
	*Sarcocystis* spp., *Eimeria* spp., *Babesia* spp., *Cryptosporidium* spp.	(Schares *et al.* 2000)
	*Leishmania infantum*	(Hosseininejad *et al.* 2010)
Antigen-capture indirect (polyclonal antiserum)	*T. gondii, Sarcocystis* spp., *Eimeria* spp., *Babesia* spp., *Cryptosporidium* spp.	(Schares *et al.* 1999*b*)
Antigen-capture competitive ELISA (Mabs 5B6-25 and 4A4-2)	*T. gondii, Sarcocystis* spp.	(Baszler *et al.* 2001)
Competitive ELISA (4A4-2)	*T. gondii*	(Baszler *et al.* 1996)^e^
	*Sarcocystis* spp.	(Baszler *et al.* 1996)^e^
Competitive ELISA (rabbit polyclonal antibody)	*T. gondii, Sarcocystis* spp., *Eimeria* spp., *Babesia* spp.	(McGarry *et al*. 2000)

a Cross-reactions observed in serum dilutions lower than the cutoff.

b Rabbit sera against *T. gondii* and *S. cruzi* do not react in WB with ISCOM antigen.

c MAbs against ISCOM antigens do not recognize *T. gondii* in WB.

d Mouse serum immunized with ISCOM antigen does not recognize *T. gondii*.

e Some sera showed elevated levels of cross-reactions in WB.

Nowadays it is becoming clear that there is a need to characterize new molecular markers that are species-specific for *T. gondii* and *N. caninum* for the development of new diagnostic tools (Zhang *et al.* 2011; Regidor-Cerrillo *et al.* 2015). In this context, the identification of cross-reactive and species-specific antigens between *N. caninum* and *T. gondii* tachyzoites is mandatory and the proteomics approach constitutes an appropriate strategy for this purpose (Zhang *et al.* 2011). These authors demonstrated the usefulness of proteomics to immuno-screen for cross-reactive or species-specific antigens from both parasites. Moreover, they showed that there was significant homology in the antigenic proteome profiles between the two parasites (Zhang *et al.* 2011). Taking together, these findings shed light on the process to design new diagnostic tools in order to avoid cross-reactivity between *N. caninum* and *T. gondii* diagnostic tests.

The characterization of cross-reactive antigens between *T. gondii* and *N. caninum* has been achieved in some studies. An NTPase identified in *N. caninum* tachyzoites was antigenically cross-reactive to the NTPases of *T. gondii* (Asai *et al.* 1998). Protein disulphide isomerase (PDI), heat-shock protein 70 (HSP70) and ribosomal protein P1 (RP1), were identified as cross-reactive antigens between the two parasites even when using MAbs, due to the high degree of homology among these parasite components (Liao *et al.* 2005*a*). Zhang *et al.* (2007*a*) demonstrated that antibodies raised against the apical membrane antigen 1 of *T. gondii* (TgAMA 1) also recognize recombinant NcAMA 1. The ribosomal phosphoprotein (P0) was shown to be a cross-reactive antigen between *T. gondii* and *N. caninum* (Zhang *et al.* 2007*b*); antibodies raised against rNcPO inhibited the growth of both *T. gondii* and *N. caninum* tachyzoites. A protease with 42 kDa was localized in the rhoptry of *T. gondii* by means of a MAb; this MAb also reacted to a 42 kDa protein in *N. caninum*, which was also localized in the rhoptry of this parasite (Ahn *et al.* 2001).

The genome of *N. caninum* (NC-Liverpool strain) was compared with the available genome of the ME-49 strain of *T. gondii* (Reid *et al.* 2012). The authors found a high synteny between the two genomes and pointed out that most divergences occurred within the SRS antigens. Transcriptome analysis suggested that *N. caninum* uses fewer SRS antigens than *T. gondii* (Reid *et al.* 2012). Therefore, selecting those species-specific parasitic surface antigens for the establishment of serologic tests, such as SRSs, may favour the specificity of these tests.

### Serological tests for N. caninum and cross-reactivity with Sarcocystis spp., B. besnoiti, N. hughesi and Hammondia spp.

Sera from *Sarcocystis* spp.-infected cattle have been shown to cross-react with several *N. caninum* antigens by WB (Baszler *et al*. 1996). However, antibodies against *Sarcocystis* spp. did not cross-react with *N. caninum*-immunodominant antigens (19, 29, 30 and 37 kDa) (Bjerkas *et al*. 1994).

Sera from calves that were experimentally infected with *Sarcocystis* spp. tested positive by a conventional ELISA using crude *N. caninum* antigen (Dubey *et al*. 1996); the same sera tested negative by *N. caninum* IFAT. In contrast, positive sera against several *Sarcocystis* spp. (*S. cruzi, S. hirsuta, S. hominis* and *S. neurona*) did not result in positive reactions in ELISAs based on *N. caninum*-selected antigens (ISCOM, whole-fixed tachyzoites, affinity-purified and recombinant) (Björkman *et al*. 1994; Baszler *et al.* 1996, 2001; Lally *et al*. 1996; Schares *et al*. 2000; Howe *et al*. 2002). Serologic cross-reactivity between infections caused by *N. caninum* and *Sarcocystis* spp. seems to be neglegible when *N. caninum*-specific antigens are employed (Table 3).

*Besnoitia besnoiti* and *N. caninum* have cattle as their major hosts and may co-infect a high proportion of animals in regions where these parasites are endemic (Jacquiet *et al.* 2010). Serologic tests such as indirect ELISA and IFAT had been developed over several decades for *B. besnoiti* antibodies (Frank *et al.* 1970; Neuman, 1972; Janitschke *et al.* 1984; Shkap *et al*. 1984), but at that time, the closely related parasite *N. caninum* was unknown, so the specificity of those tests could not have been ascertained. Later, it was shown that sera from *N. caninum*-positive cattle and gerbils recognized *B. besnoiti* antigens by IFAT when a less stringent cutoff (1:64) was used (Shkap *et al.* 2002); the authors also observed that these animal sera reacted against two bands of *B. besnoiti* antigens under reducing conditions by WB. In another study, a more stringent cutoff (1:200) for *B. besnoiti* IFAT did not show cross-reactions with sera from *N. caninum*-infected animals, whereas serum dilutions of 1:100 showed some level of cross-reactivity (Schares *et al.* 2010).

*Besnoitia besnoiti* WBs and indirect ELISAs were developed for detection of antibodies in cattle and were tested for cross-reactivity with sera from animals infected with *N. caninum* and *T. gondii* (Cortes *et al.* 2006; Fernandez-Garcia *et al.* 2010; Schares *et al.* 2011). Cross-reactivity with *N. caninum* was observed in the tested ELISAs and WBs, especially with animals exhibiting high antibody titres for *N. caninum*. A novel ELISA for *B. besnoiti* was developed with affinity-purified antigens of confirmed relevance, mostly localized on the surface of tachyzoites. This ELISA has shown a lower degree of cross-reactivity with *N. caninum* (Schares *et al.* 2013). A recent study demonstrated that cattle exhibiting high antibody levels to *N. caninum* and/or *Sarcocystis* spp. presented a higher number of false-positive reactions for *B. besnoiti* by an in-house ELISA (Garcia-Lunar *et al.* 2015); thus, infection with *Sarcocystis* spp. and *N. caninum* may augment cross-reactions with *B. besnoiti*.

No serologic cross-reactivity has been observed between *N. caninum* and *H. heydorni*. A few serum samples from mice, dog and sheep that were infected with *H. heydorni* did not cross-react with *N. caninum* antigens by IFAT, ELISA or WB (Nishikawa *et al.* 2002; Gondim *et al.* 2015). However, further studies using larger numbers of sera from *H. heydorni*-infected animals are necessary to confirm the absence of cross-reactivity between *N. caninum* and *H. heydorni*.

*Neospora hughesi* was proposed as a new species based on seven nucleotide differences in the internal transcribed spacer 1 (ITS1) of the rDNA, as well as on ultrastructural and antigenic differences when compared with *N. caninum* (Marsh *et al.* 1998). The acceptance of *N. hughesi* as a new species was reinforced by differences observed in amino acid sequences of two surface antigens (SAG1 and SRS2) to those from *N. caninum* (Marsh *et al.* 1999). However, polyclonal serum from a *N. caninum*-infected rabbit recognized NcSAG1, NhSAG1, NcSRS2 and NhSRS2 by immunoblot (Marsh *et al.* 1999). Differences also have been found in gene sequences between the dense granule proteins GRA6 and GRA7 of *N. caninum* and *N. hughesi*, although polyclonal serum to *N. caninum* recognized GRA6 and GRA7 antigens from both *N. caninum* and *N. hughesi* by immunoblot (Walsh *et al.* 2001).

An ELISA for *N. hughesi* antibodies was developed using a recombinant NhSAG1 as antigen (Hoane *et al.* 2005); animals infected with *N. hughesi* presented a higher antibody reactivity to rNhSAG1 than to rNcSAG1, but the test was not able to unambiguously differentiate infections caused by *N. hughesi* or *N. caninum*. In another study, pre- and post-infection sera from dog and cattle that were experimentally inoculated with *N. caninum* were tested simultaneously by IFAT using tachyzoites of *N. caninum* or *N. hughesi* (Gondim *et al.* 2009); all sera that tested positive for *N. caninum* also reacted with *N. hughesi* tachyzoites, although the antibody titres for *N. hughesi* IFAT were slightly lower as compared with the IFAT for *N. caninum*. To date, infections caused by *N. caninum* and *N. hughesi* cannot be serologically discriminated. As horses may be infected by both *N. caninum* and *N. hughesi* (Marsh *et al*. 1999; Pitel *et al.* 2003; Veronesi *et al.* 2008), a species-specific serologic test for equines is desired.

### Serological tests based on chimeric antigens, synthetic peptides and stage-specific antigens

#### Chimeric antigens

Almost 30 years ago, the production of recombinant polypeptides derived from genes encoding *T. gondii* antigens revolutionized the search for more efficient serologic methods (Johnson *et al.* 1989; Johnson and Illana, 1991). The development of ELISAs based on a mixture of recombinant antigens, rather than the use of a single recombinant protein, has been presumed to increase the sensitivity of the ELISA for human sera, while maintaining the desired specificity (Johnson *et al.* 1992; Aubert *et al.* 2000). ELISAs containing a mixture of recombinant antigens were also tested for antibodies against *T. gondii* in sheep and cats (Tenter *et al.* 1992).

A chimeric antigen is the fusion of gene fragments constructed as a single gene and expressed to form a hybrid protein (Yang *et al.* 2004). The use of chimeric antigens for *T. gondii* serology is promising. Chimeric proteins are usually larger than single recombinant antigens resulting in a better binding to microtitre plates. In addition, as a chimeric antigen preparation consists of a single antigen, it may be easier to standardize than mixtures of recombinant antigens (Beghetto *et al.* 2006; Lau *et al.* 2011; Holec-Gasior *et al.* 2012*a*).

Several chimeric antigens have been tested by WB or ELISA for the detection of human antibodies against *T. gondii*. Among the developed chimeric antigens tested by serology, are preparations including parts of well-characterized immunodominant proteins, such as SAG1, SAG2, MIC1, MIC2, MIC3, MAG1, M2AP, GRA1, GRA2, GRA3 and ROP1 (Beghetto *et al.* 2006; Lau *et al.* 2011; Holec-Gasior *et al.* 2012*a*, *b*; Ferra *et al.* 2015*a*). The standardization of a chimeric-antigen-based test for *T. gondii* antibodies depends on various factors, including an optimal selection of antigens and proper expression of all desired epitopes. In addition to humans, a recent paper reports the first trial of chimeric antigens employed for *T. gondii* serology in farm animals (horses, swine and sheep) (Ferra *et al.* 2015*b*); the authors validated their antigen with more than 400 sera and also included 15 sera, which were serologically positive for *N. caninum* but negative for *T. gondii*. It is interesting to note that each animal species responded differently to the chimeric-antigen preparations used in the ELISAs, but the SAG2–GRA1–ROP1_L_ construct reached the best overall specificity and sensitivity for the three tested species (Ferra *et al.* 2015*b*). Despite progress in the development of chimeric antigens for *T. gondii* serology, in particular for humans, inadequate information is available about the serologic cross-reactivity potential of those tests with sera from animals infected with other Toxoplasmatinae parasites.

#### Synthetic peptides, serotyping and stage-specific antigens

The combination of molecular engineering and chemical synthesis of antigens has been applied for the development of serological techniques with improved sensitivity and specificity. Synthetic peptides representing several epitopes of numerous antigens have been used in microarray assays for serotyping of viral and bacterial diseases (Neuman de Vegvar *et al.* 2003; Nahtman *et al.* 2007).

In *T. gondii* infections, the humoral response has been demonstrated to be partially strain-specific. In one study, MAbs produced against SAG2A from naturally infected mice recognized the surface antigens encoded by the SAG2 allele of type I and III strains, but not of type II strains (Parmley *et al.* 1994). Another study identified a MAb showing differences in the recognition of type II and III strains (Bohne *et al.* 1993). Kong *et al.* (2003) screened nucleotide sequences from types I, II and III of *T. gondii* for the identification of polymorphic regions from genes coding selected antigens. Allele-specific peptides were synthetized and screened by ELISA using sera from mice and humans. Synthetic peptides based on SAG2A, GRA3, GRA6 and GRA7 were able to discriminate type II from non-type II infections (Kong *et al.* 2003). In subsequent studies, serotyping for *T. gondii* infections using new recombinant polypeptides or new or improved synthetic peptides, as well as target populations from different regions, have been performed by ELISAs (Peyron *et al.* 2006; Sousa *et al.* 2008, 2009). In two studies, peptide-microarrays were validated to discriminate the serological responses against clonal-type *T. gondii* strains in samples from humans (Maksimov *et al.* 2012*b*) and cats (Maksimov *et al.* 2013). The latter study used sera from experimentally infected cats for validation and showed significant type-specific differences in the IgG response against the tested peptide panel. However, in many peptides, reactions were not clonal type-specific (Maksimov *et al*. 2013).

A peptide microarray was developed and validated for serotyping *T. gondii* infections in humans, aiming to differentiate between different manifestations of *T. gondii* infection (Maksimov *et al.* 2012*a*). Thirty eight *T. gondii* synthetic peptides, consisting of 18 peptides characterized in previous studies, and 20 novel peptides, predicted by bioinformatics approach, were tested to differentiate acute, latent and ocular infections. Some peptides based on dense granule and microneme antigens (GRA2-28, MIC3-282 and MIC3-191) showed promising results for differentiation between acute and latent infections (Maksimov *et al.* 2012*a*).

The use of synthetic peptides for *T. gondii* serotyping may have a great potential for discriminating between *T. gondii* infections and infections caused by other parasite species expected to induce some degree of serologic cross-reactivity. Of course, one important prerequisite is that peptides applied in such tests are not specific for particular clonal-types or genotypes of *T. gondii* or related parasite species. In addition, potential cross-reactivities need to be addressed; for instance, synthetic peptides considered to react specifically with antibodies generated by *T. gondii* infections need to be tested with sera from animals exposed to *H. hammondi*, as this parasite has a very close genetic relationship to the former (Walzer *et al.* 2013, 2014). The use of *T. gondii*-specific peptides for diagnosis in animals should also be tested with *N. caninum*, which may cross-react, as shown in Table 3. To our knowledge, there are no published studies using synthetic peptides to differentiate infections in animals cause by *T. gondii, H. hammondi* or *N. caninum*.

Stage-specific antigens (native or recombinant) from *T. gondii* have been investigated for decades by several research groups, including oocysts and sporozoites which can be produced in cats (Kasper *et al.* 1984; Ferguson *et al.* 2000; Dumetre and Darde, 2007; Possenti *et al.* 2010, 2013; Bushkin *et al.* 2012; Fritz *et al.* 2012). In contrast, antigens from *Neospora* spp. are mostly obtained from tachyzoites. Only small numbers of studies have produced *N. caninum* tissue cysts in cell culture or in animals (Weiss *et al.* 1999; Tunev *et al.* 2002; Risco-Castillo *et al.* 2004; Vonlaufen *et al.* 2004), or have induced oocyst production in canid definitive hosts (McAllister *et al.* 1998; Schares *et al.* 2001; Gondim *et al.* 2002).

The identification of the first bradyzoite-specific gene of *N. caninum* (Nc*SAG4*), an orthologue to Tg*SAG4*, allowed the production and characterization of the recombinant protein rNcSAG4 (Fernandez-Garcia *et al*. 2006). This antigen has been applied for the development of a stage-specific ELISA for bovine neosporosis (Aguado-Martinez *et al.* 2008).

The production of *Hammondia* spp. antigens is even more difficult, because there is no permanent culture for these parasites (Riahi *et al.* 1995; Schares *et al.* 2003; Gondim *et al.* 2015). Therefore, most antigens from *Hammondia* spp. are derived from oocysts or from parasite cysts from intermediate hosts (Riahi *et al.* 1998, 1999, 2000; Walzer *et al.* 2013). Generation of recombinant antigens from *Hammondia* spp. is lacking. It would enable studies on exposure of animals and humans to these parasites, as well as on serologic cross-reactivity with related protozoa.

A serologic test for humans based on a *T. gondii* sporozoite-specific protein has been evaluated and seems to represent a promising method to discriminate between oocyst-induced infections from those transmitted by ingestion of infected meat (Hill *et al.* 2011), which is an important consideration in epidemiological investigations. This study was the first to use a sporozoite protein, called *T. gondii* embryogenesis-related protein (TgERP) in a serologic test for *T. gondii*. In a recent study another sporozoite-derived protein called CCp5A was successfully used in an ELISA to identify oocyst-infected animals (Santana *et al.* 2015); this test was able to identify antibodies in sera from humans, pigs, mice and chickens that were naturally or experimentally infected with *T. gondii* oocysts and discriminate between animals infected from ingestion of oocysts or by carnivorism. Further investigations are necessary for validation and to confirm whether the sporozoite proteins TgERP (Hill *et al.* 2011) and CCp5A (Santana *et al.* 2015) cross-react with sporozoite proteins derived from *Hammondia* spp. and *Neospora* spp. It is also important to test whether low infectious dose with oocysts will elicit detectable antibodies to TgERP or CCp5A.

Type or stage-specific antigens have a wide spectrum of applications, including investigations of infection outbreaks and identification of risk factors in selected populations (e.g. ingestion of oocyst or tissue cysts). Those antigens should be used with caution and only after rigorous validation in epidemiological studies.

## Concluding remarks

Most currently available serologic tests for *T. gondii* may show some level of cross-reactivity with related coccidia, in particular with *H. hammondi* and *N. caninum*. Serological cross-reactivity between *T. gondii* and *N. caninum* has been observed when crude antigen ELISAs are employed. Several ELISAs based on recombinant species-specific antigens did not present cross-reactivity with *N. caninum*. Therefore, *T. gondii* shares more surface antigens with *H. hammondi* than with *N. caninum*.

When detecting *T. gondii* antibodies in animals, IFAT seems to be more specific than the ELISAs based on crude antigen. Since *N. caninum* is not considered to be a human pathogen, the major concern regarding serologic cross-reactivity would be potential exposure to *H. hammondi*, although it is unknown whether this parasite is able to induce infection in humans.

Antibodies from cattle infected with *B. besnoiti* cross-react with *N. caninum* antigens by IFAT in serum dilutions lower than the recommended cutoff (1:200). Novel *B. besnoiti* ELISAs (e.g. ELISA based on purified surface antigens) showed a lower degree of cross-reactivity with sera from cattle infected with *N. caninum* or other related parasites. Antibodies against bovine *Sarcocystis* sp. present negligible cross-reactions with *N. caninum*-immunodominant antigens. The development of ELISAs for *N. caninum* antibodies based on chimeric peptides specific for the parasite seems to be promising. Chimeric antigens would enable a better standardization of serologic tests, as a single protein is used. Moreover, chimeric antigens would potentially present higher sensitivity than single recombinant antigens. Despite the close phylogenetic relationship between *N. caninum* and *H. heydorni*, no evidence of serologic cross-reactivity between these protozoa has been confirmed to date. *Toxoplasma gondii* infections cause more confusion with *N. caninum* serology than those infections induced by *H. heydorni. Neospora caninum* and *N. hughesi* infections cannot be serologically differentiated, because currently available serologic tests are genus rather than species-specific for these protozoa.

## References

[ref1] Aguado-Martinez A., Alvarez-Garcia G., Fernandez-Garcia A., Risco-Castillo V., Arnaiz-Seco I., Rebordosa-Trigueros X., Navarro-Lozano V. and Ortega-Mora L. M. (2008). Usefulness of rNcGRA7- and rNcSAG4-based ELISA tests for distinguishing primo-infection, recrudescence, and chronic bovine neosporosis. Veterinary Parasitology 157, 182–195. 18814972 10.1016/j.vetpar.2008.08.002

[ref2] Ahn H. J., Song K. J., Son E. S., Shin J. C. and Nam H. W. (2001). Protease activity and host cell binding of the 42-kDa rhoptry protein from *Toxoplasma gondii* after secretion. Biochemical *and* Biophysical Research Communications 287, 630–635. 11563841 10.1006/bbrc.2001.5637

[ref3] Alvarez-Garcia G., Pereira-Bueno J., Gomez-Bautista M. and Ortega-Mora L. M. (2002). Pattern of recognition of *Neospora caninum* tachyzoite antigens by naturally infected pregnant cattle and aborted foetuses. Veterinary Parasitology 107, 15–27. 12072210 10.1016/s0304-4017(02)00091-2

[ref4] Alvarez-Garcia G., Pitarch A., Zaballos A., Fernandez-Garcia A., Gil C., Gomez-Bautista M., Aguado-Martinez A. and Ortega-Mora L. M. (2007). The NcGRA7 gene encodes the immunodominant 17 kDa antigen of *Neospora caninum*. Parasitology 134, 41–50. 17032479 10.1017/S0031182006001284

[ref5] Alvarez-Garcia G., Frey C. F., Mora L. M. and Schares G. (2013). A century of bovine besnoitiosis: an unknown disease re-emerging in Europe. Trends in Parasitology 29, 407–415. 23830145 10.1016/j.pt.2013.06.002

[ref6] Anderson M. L., Blanchard P. C., Barr B. C., Dubey J. P., Hoffman R. L. and Conrad P. A. (1991). *Neospora*-like protozoan infection as a major cause of abortion in California dairy cattle. Journal of the American Veterinary Medical Association 198, 241–244. 2004983

[ref7] Andrews C. D., Fayer R. and Dubey J. P. (1990). Continuous *in vitro* cultivation of *Sarcocystis cruzi*. Journal of Parasitol 76, 254–255. 2108236

[ref8] Araujo F. G., Dubey J. P. and Remington J. S. (1984). Antigenic similarity between the coccidian parasites *Toxoplasma gondii* and *Hammondia hammondi*. Journal of Protozoology 31, 145–147. 6204042 10.1111/j.1550-7408.1984.tb04304.x

[ref9] Asai T., Howe D. K., Nakajima K., Nozaki T., Takeuchi T. and Sibley L. D. (1998). *Neospora caninum*: tachyzoites express a potent type-I nucleoside triphosphate hydrolase. Experimental Parasitology 90, 277–285. 9806873 10.1006/expr.1998.4346

[ref10] Atkinson R. A., Cook R. W., Reddacliff L. A., Rothwell J., Broady K. W., Harper P. and Ellis J. T. (2000). Seroprevalence of *Neospora caninum* infection following an abortion outbreak in a dairy cattle herd. Australian Veterinary Journal 78, 262–266. 10840574 10.1111/j.1751-0813.2000.tb11752.x

[ref12] Aubert D., Maine G. T., Villena I., Hunt J. C., Howard L., Sheu M., Brojanac S., Chovan L. E., Nowlan S. F. and Pinon J. M. (2000). Recombinant antigens to detect *Toxoplasma gondii*-specific immunoglobulin G and immunoglobulin M in human sera by enzyme immunoassay. Journal of Clinical Microbiology 38, 1144–1150. 10699010 10.1128/jcm.38.3.1144-1150.2000 PMC86359

[ref13] Barr B. C., Conrad P. A., Dubey J. P. and Anderson M. L. (1991). *Neospora*-like encephalomyelitis in a calf: pathology, ultrastructure, and immunoreactivity. Journal of Veterinary Diagnostic Investigation 3, 39–46. 2039786 10.1177/104063879100300109

[ref14] Barta J. R. and Dubey J. P. (1992). Characterization of anti-*Neospora caninum* hyperimmune rabbit serum by western blot analysis and immunoelectron microscopy. Parasitology Research 78, 689–694. 1480607 10.1007/BF00931522

[ref15] Basso W., Schares G., Gollnick N. S., Rutten M. and Deplazes P. (2011). Exploring the life cycle of *Besnoitia besnoiti* – experimental infection of putative definitive and intermediate host species. Veterinary Parasitology 178, 223–234. 21310538 10.1016/j.vetpar.2011.01.027

[ref16] Baszler T. V., Knowles D. P., Dubey J. P., Gay J. M., Mathison B. A. and McElwain T. F. (1996). Serological diagnosis of bovine neosporosis by *Neospora caninum* monoclonal antibody-based competitive inhibition enzyme-linked immunosorbent assay. Journal of Clinical Microbiology 34, 1423–1428. 8735092 10.1128/jcm.34.6.1423-1428.1996 PMC229036

[ref17] Baszler T. V., Adams S., Vander-Schalie J., Mathison B. A. and Kostovic M. (2001). Validation of a commercially available monoclonal antibody-based competitive-inhibition enzyme-linked immunosorbent assay for detection of serum antibodies to *Neospora caninum* in cattle. Journal of Clinical Microbiology 39, 3851–3857. 11682497 10.1128/JCM.39.11.3851-3857.2001 PMC88454

[ref18] Beghetto E., Spadoni A., Bruno L., Buffolano W. and Gargano N. (2006). Chimeric antigens of *Toxoplasma gondii*: toward standardization of toxoplasmosis serodiagnosis using recombinant products. Journal of Clinical Microbiology 44, 2133–2140. 16757610 10.1128/JCM.00237-06 PMC1489449

[ref19] Bela S. R., Oliveira Silva D. A., Cunha-Junior J. P., Pirovani C. P., Chaves-Borges F. A., Reis de Carvalho F., Carrijo de Oliveira T. and Mineo J. R. (2008). Use of SAG2A recombinant *Toxoplasma gondii* surface antigen as a diagnostic marker for human acute toxoplasmosis: analysis of titers and avidity of IgG and IgG1 antibodies. Diagnostic Microbiology and Infectious Disease 62, 245–254. 18703303 10.1016/j.diagmicrobio.2008.05.017

[ref20] Benetti A. H., Schein F. B., dos Santos T. R., Toniollo G. H., da Costa A. J., Mineo J. R., Lobato J., de Oliveira Silva D. A. and Gennari S. M. (2009). Inquiry of antibodies anti-*Neospora caninum* in dairy cattle, dogs and rural workers of the south-west region of Mato Grosso State. Revista Brasileira de Parasitologia Veterinária 18(Suppl 1), 29–33. 10.4322/rbpv.018e1005 20040187

[ref21] Beverley J. K. and Beattie C. P. (1952). Standardization of the dye test for toxoplasmosis. Journal of Clinical Pathology 5, 350–353. 13011224 10.1136/jcp.5.4.350 PMC1023675

[ref22] Bjerkas I., Mohn S. F. and Presthus J. (1984). Unidentified cyst-forming sporozoon causing encephalomyelitis and myositis in dogs. Zeitschrift für Parasitenkunde 70, 271–274. 6426185 10.1007/BF00942230

[ref23] Bjerkas I., Jenkins M. C. and Dubey J. P. (1994). Identification and characterization of *Neospora caninum* tachyzoite antigens useful for diagnosis of neosporosis. Clinical and Diagnostic Laboratory Immunology 1, 214–221. 7496948 10.1128/cdli.1.2.214-221.1994 PMC368230

[ref24] Björkman C. and Hemphill A. (1998). Characterization of *Neospora caninum* iscom antigens using monoclonal antibodies. Parasite Immunology 20, 73–80. 9572050 10.1046/j.1365-3024.1998.00127.x

[ref25] Björkman C. and Lunden A. (1998). Application of iscom antigen preparations in ELISAs for diagnosis of *Neospora* and *Toxoplasma* infections. International Journal for Parasitology 28, 187–193. 9504345 10.1016/s0020-7519(97)00174-4

[ref26] Björkman C., Lunden A., Holmdahl J., Barber J., Trees A. J. and Uggla A. (1994). *Neospora caninum* in dogs: detection of antibodies by ELISA using an iscom antigen. Parasite Immunology 16, 643–648. 7708430 10.1111/j.1365-3024.1994.tb00320.x

[ref27] Björkman C. and Uggla A. (1999). Serological diagnosis of *Neospora caninum* infection. International Journal for Parasitology 29, 1497–1507. 10608435 10.1016/s0020-7519(99)00115-0

[ref28] Björkman C., Holmdahl O. J. and Uggla A. (1997). An indirect enzyme-linked immunoassay (ELISA) for demonstration of antibodies to *Neospora caninum* in serum and milk of cattle. Veterinary Parasitology 68, 251–260. 9066070 10.1016/s0304-4017(96)01076-x

[ref29] Blagburn B. L., Lindsay D. S., Swango L. J., Pidgeon G. L. and Braund K. G. (1988). Further characterization of the biology of *Hammondia heydorni*. Veterinary Parasitology 27, 193–198. 3369072 10.1016/0304-4017(88)90033-7

[ref30] Bohne W., Gross U. and Heesemann J. (1993). Differentiation between mouse-virulent and -avirulent strains of *Toxoplasma gondii* by a monoclonal antibody recognizing a 27-kilodalton antigen. Journal of Clinical Microbiology 31, 1641–1643. 8315008 10.1128/jcm.31.6.1641-1643.1993 PMC265596

[ref31] Burg J. L., Perelman D., Kasper L. H., Ware P. L. and Boothroyd J. C. (1988). Molecular analysis of the gene encoding the major surface antigen of *Toxoplasma gondii*. Journal of Immunology 141, 3584–3591. 3183382

[ref32] Bushkin G. G., Motari E., Magnelli P., Gubbels M. J., Dubey J. P., Miska K. B., Bullitt E., Costello C. E., Robbins P. W. and Samuelson J. (2012). Beta-1,3-glucan, which can be targeted by drugs, forms a trabecular scaffold in the oocyst walls of *Toxoplasma* and *Eimeria*. MBio 3. doi: 10.1128/mBio.00258-12. PMC3518913 23015739

[ref33] Buxton D. (1998). Protozoan infections (*Toxoplasma gondii, Neospora caninum* and *Sarcocystis* spp.) in sheep and goats: recent advances. Veterinary Research 29, 289–310. 9689743

[ref34] Carreno R. A., Schnitzler B. E., Jeffries A. C., Tenter A. M., Johnson A. M. and Barta J. R. (1998). Phylogenetic analysis of coccidia based on 18S rDNA sequence comparison indicates that *Isospora* is most closely related to *Toxoplasma* and *Neospora*. Journal of Eukaryotic Microbiology 45, 184–188. 9561772 10.1111/j.1550-7408.1998.tb04523.x

[ref35] Carvalho F. R., Silva D. A., Cunha-Junior J. P., Souza M. A., Oliveira T. C., Bela S. R., Faria G. G., Lopes C. S. and Mineo J. R. (2008). Reverse enzyme-linked immunosorbent assay using monoclonal antibodies against SAG1-related sequence, SAG2A, and p97 antigens from *Toxoplasma gondii* to detect specific immunoglobulin G (IgG), IgM, and IgA antibodies in human sera. Clinical and Vaccine Immunology 15, 1265–1271. 18562566 10.1128/CVI.00069-08 PMC2519308

[ref36] Cesbron-Delauw M. F., Tomavo S., Beauchamps P., Fourmaux M. P., Camus D., Capron A. and Dubremetz J. F. (1994). Similarities between the primary structures of two distinct major surface proteins of *Toxoplasma gondii*. Journal of Biological Chemistry 269, 16217–16222. 8206924

[ref37] Chahan B., Gaturaga I., Huang X., Liao M., Fukumoto S., Hirata H., Nishikawa Y., Suzuki H., Sugimoto C., Nagasawa H., Fujisaki K., Igarashi I., Mikami T. and Xuan X. (2003). Serodiagnosis of *Neospora caninum* infection in cattle by enzyme-linked immunosorbent assay with recombinant truncated NcSAG1. Veterinary Parasitology 118, 177–185. 14729165 10.1016/j.vetpar.2003.10.010

[ref38] Christie E. and Dubey J. P. (1977). Cross-immunity between *Hammondia* and *Toxoplasma* infections in mice and hamsters. Infection and Immunity 18, 412–415. 411757 10.1128/iai.18.2.412-415.1977 PMC421248

[ref39] Cortes H. C., Nunes S., Reis Y., Staubli D., Vidal R., Sager H., Leitao A. and Gottstein B. (2006). Immunodiagnosis of *Besnoitia besnoiti* infection by ELISA and Western blot. Veterinary Parasitology 141, 216–225. 16822616 10.1016/j.vetpar.2006.05.023

[ref40] Cortes H., Leitao A., Gottstein B. and Hemphill A. (2014). A review on bovine besnoitiosis: a disease with economic impact in herd health management, caused by *Besnoitia besnoiti* (Franco and Borges,). Parasitology 141, 1406–1417. 24694568 10.1017/S0031182014000262

[ref41] Cunha-Junior J. P., Silva D. A., Silva N. M., Souza M. A., Souza G. R., Prudencio C. R., Pirovani C. P., Cezar M. C. J., Barbosa B. F., Goulart L. R. and Mineo J. R. (2010). A4D12 monoclonal antibody recognizes a new linear epitope from SAG2A *Toxoplasma gondii* tachyzoites, identified by phage display bioselection. Immunobiology 215, 26–37. 19261354 10.1016/j.imbio.2009.01.008

[ref42] Desmonts G. and Remington J. S. (1980). Direct agglutination test for diagnosis of *Toxoplasma* infection: method for increasing sensitivity and specificity. Journal of Clinical Microbiology 11, 562–568. 7000807 10.1128/jcm.11.6.562-568.1980 PMC273461

[ref43] Dubey J. P. (1981). Protective immunity against clinical toxoplasmosis in dairy goats vaccinated with *Hammondia hammondi* and *Hammondia heydorni*. American Journal of Veterinary Research 42, 2068–2070. 7340577

[ref44] Dubey J. P. and Lindsay D. S. (2006). Neosporosis, toxoplasmosis, and sarcocystosis in ruminants. Veterinary Clinics of North America Food Animal Practice 22, 645–671. 17071358 10.1016/j.cvfa.2006.08.001

[ref45] Dubey J. P. and Sreekumar C. (2003). Redescription of *Hammondia hammondi* and its differentiation from *Toxoplasma gondii*. International Journal for Parasitology 33, 1437–1453. 14572507 10.1016/s0020-7519(03)00141-3

[ref46] Dubey J. P., Carpenter J. L., Speer C. A., Topper M. J. and Uggla A. (1988 *a*). Newly recognized fatal protozoan disease of dogs. Journal of the American Veterinary Medical Association 192, 1269–1285. 3391851

[ref47] Dubey J. P., Hattel A. L., Lindsay D. S. and Topper M. J. (1988 *b*). Neonatal *Neospora caninum* infection in dogs: isolation of the causative agent and experimental transmission. Journal of the American Veterinary Medical Association 193, 1259–1263. 3144521

[ref48] Dubey J. P., Lindsay D. S., Adams D. S., Gay J. M., Baszler T. V., Blagburn B. L. and Thulliez P. (1996). Serologic responses of cattle and other animals infected with *Neospora caninum*. American Journal of Veterinary Research 57, 329–336. 8669764

[ref49] Dubey J. P., Jenkins M. C., Rajendran C., Miska K., Ferreira L. R., Martins J., Kwok O. C. and Choudhary S. (2011). Gray wolf (*Canis lupus*) is a natural definitive host for *Neospora caninum*. Veterinary Parasitology 181, 382–387. 21640485 10.1016/j.vetpar.2011.05.018

[ref50] Dubey J. P., More G., van Wilpe E., Calero-Bernal R., Verma S. K. and Schares G. (2016). *Sarcocystis rommeli*, n. sp. (Apicomplexa: Sarcocystidae) from Cattle (*Bos taurus*) and its Differentiation from *Sarcocystis hominis*. Journal of Eukaryotic Microbiology 63, 62–68. 26111603 10.1111/jeu.12248

[ref51] Dumetre A. and Darde M. L. (2007). Detection of *Toxoplasma gondii* in water by an immunomagnetic separation method targeting the sporocysts. Parasitology Research 101, 989–996. 17530288 10.1007/s00436-007-0573-0

[ref52] Ferguson D. J., Brecht S. and Soldati D. (2000). The microneme protein MIC4, or an MIC4-like protein, is expressed within the macrogamete and associated with oocyst wall formation in *Toxoplasma gondii*. International Journal for Parasitology 30, 1203–1209. 11027789 10.1016/s0020-7519(00)00096-5

[ref53] Fernandez-Garcia A., Risco-Castillo V., Zaballos A., Alvarez-Garcia G. and Ortega-Mora L. M. (2006). Identification and molecular cloning of the *Neospora caninum* SAG4 gene specifically expressed at bradyzoite stage. Molecular and Biochemical Parasitology 146, 89–97. 16303187 10.1016/j.molbiopara.2005.08.019

[ref54] Fernandez-Garcia A., Alvarez-Garcia G., Risco-Castillo V., Aguado-Martinez A., Marcen J. M., Rojo-Montejo S., Castillo J. A. and Ortega-Mora L. M. (2010). Development and use of an indirect ELISA in an outbreak of bovine besnoitiosis in Spain. Veterinary Record 166, 818–822. 20581359 10.1136/vr.b4874

[ref55] Ferra B., Holec-Gasior L. and Kur J. (2015 *a*). A new *Toxoplasma gondii* chimeric antigen containing fragments of SAG2, GRA1, and ROP1 proteins-impact of immunodominant sequences size on its diagnostic usefulness. Parasitology Research 114, 3291–3299. 26055987 10.1007/s00436-015-4552-6 PMC4537703

[ref56] Ferra B., Holec-Gasior L. and Kur J. (2015 *b*). Serodiagnosis of *Toxoplasma gondii* infection in farm animals (horses, swine, and sheep) by enzyme-linked immunosorbent assay using chimeric antigens. Parasitology International 64, 288–294. 25817245 10.1016/j.parint.2015.03.004

[ref57] Frank M., Klinger I. and Pipano E. (1970). The presence of antibody against *Besnoitia besnoiti* in dairy and beef cattle. Journal of Protozoology 17, 31.

[ref58] Frenkel J. K. and Dubey J. P. (1975). *Hammondia hammondi* gen. nov., sp.nov., from domestic cats, a new coccidian related to *Toxoplasma* and *Sarcocystis*. Zeitschrift für Parasitenkunde 46, 3–12. 807048 10.1007/BF00383662

[ref59] Fritz H. M., Bowyer P. W., Bogyo M., Conrad P. A. and Boothroyd J. C. (2012). Proteomic analysis of fractionated *Toxoplasma* oocysts reveals clues to their environmental resistance. PLoS ONE 7, e29955. 22279555 10.1371/journal.pone.0029955 PMC3261165

[ref60] Fuchs N., Sonda S., Gottstein B. and Hemphill A. (1998). Differential expression of cell surface- and dense granule-associated *Neospora caninum* proteins in tachyzoites and bradyzoites. Journal of Parasitology 84, 753–758. 9714206

[ref61] Fulton J. D. and Turk J. L. (1959). Direct agglutination test for *Toxoplasma gondii*. Lancet 2, 1068–1069. 13825641 10.1016/s0140-6736(59)91535-1

[ref62] Garcia-Lunar P., More G., Campero L., Ortega-Mora L. M. and Alvarez-Garcia G. (2015). Anti-*Neospora caninum* and anti-*Sarcocystis* spp. specific antibodies cross-react with *Besnoitia besnoiti* and influence the serological diagnosis of bovine besnoitiosis. Veterinary Parasitology 214, 49–54. 26386830 10.1016/j.vetpar.2015.09.011

[ref63] Gaturaga I., Chahan B., Xuan X., Huang X., Liao M., Fukumoto S., Hirata H., Nishikawa Y., Takashima Y., Suzuki H., Fujisaki K. and Sugimoto C. (2005). Detection of antibodies to *Neospora caninum* in cattle by enzyme-linked immunosorbent assay with truncated NcSRS2 expressed in *Escherichia coli*. The Journal of Parasitology 91, 191–192. 15856900 10.1645/GE-267R1

[ref64] Gjerde B. (2016). Molecular characterisation of *Sarcocystis bovifelis, Sarcocystis bovini* n. sp., *Sarcocystis hirsuta* and *Sarcocystis cruzi* from cattle (*Bos taurus*) and *Sarcocystis sinensis* from water buffaloes (*Bubalus bubalis*). Parasitology Research 115, 1473–1492. 26677095 10.1007/s00436-015-4881-5

[ref65] Gjerde B. and Dahlgren S. S. (2011). *Hammondia triffittae* n. comb. of foxes (*Vulpes* spp.): biological and molecular characteristics and differentiation from *Hammondia heydorni* of dogs. Parasitology 138, 303–321. 20854709 10.1017/S0031182010001265

[ref66] Gondim L. F. P., Gao L. and McAllister M. M. (2002). Improved production of *Neospora caninum* oocysts, cyclical oral transmission between dogs and cattle, and *in vitro* isolation from oocysts. Journal of Parasitology 88, 1159–1163. 12537111 10.1645/0022-3395(2002)088[1159:IPONCO]2.0.CO;2

[ref67] Gondim L. F. P., McAllister M. M., Pitt W. C. and Zemlicka D. E. (2004). Coyotes (*Canis latrans*) are definitive hosts of *Neospora caninum*. International Journal for Parasitology 34, 159–161. 15037103 10.1016/j.ijpara.2004.01.001

[ref68] Gondim L. F., Lindsay D. S. and McAllister M. M. (2009). Canine and bovine *Neospora caninum* control sera examined for cross-reactivity using *Neospora caninum* and *Neospora hughesi* indirect fluorescent antibody tests. Journal of Parasitology 95, 86–88. 18613752 10.1645/GE-1710.1

[ref69] Gondim L. F., Meyer J., Peters M., Rezende-Gondim M. M., Vrhovec M. G., Pantchev N., Bauer C., Conraths F. J. and Schares G. (2015). *In vitro* cultivation of *Hammondia heydorni*: generation of tachyzoites, stage conversion into bradyzoites, and evaluation of serologic cross-reaction with *Neospora caninum*. Veterinary Parasitology 210, 131–140. 25887985 10.1016/j.vetpar.2015.03.028

[ref70] Gondim L. F., Wolf A., Vrhovec M. G., Pantchev N., Bauer C., Langenmayer M. C., Bohne W., Teifke J. P., Dubey J. P., Conraths F. J. and Schares G. (2016). Characterization of an IgG monoclonal antibody targeted to both tissue cyst and sporocyst walls of *Toxoplasma gondii*. Experimental Parasitology 163, 46–56. 26836446 10.1016/j.exppara.2016.01.014

[ref71] Hamidinejat H., Seifi Abad Shapouri M. R., Namavari M. M., Shayan P. and Kefayat M. (2015). Development of an indirect ELISA using different fragments of recombinant Ncgra7 for detection of *Neospora caninum* infection in cattle and water buffalo. Iranian Journal of Parasitology, 10, 69–77. 25904948 PMC4403542

[ref72] Hara O. A., Liao M., Baticados W., Bannai H., Zhang G., Zhang S., Lee E.-g., Nishikawa Y., Claveria F. G. and Igarashi M. (2006). Expression of recombinant dense granule protein 7 of *Neospora caninum* and evaluation of its diagnostic potential for canine neosporosis. Journal of Protozoology Research, 16, 34–41.

[ref73] Harkins D., Clements D. N., Maley S., Marks J., Wright S., Esteban I., Innes E. A. and Buxton D. (1998). Western blot analysis of the IgG responses of ruminants infected with *Neospora caninum* and with *Toxoplasma gondii*. Journal of Comparative Pathology 119, 45–55. 9717126 10.1016/s0021-9975(98)80070-4

[ref74] Hemphill A. and Gottstein B. (1996). Identification of a major surface protein on *Neospora caninum* tachyzoites. Parasitology Research 82, 497–504. 8832729 10.1007/s004360050152

[ref75] Hemphill A., Felleisen R., Connolly B., Gottstein B., Hentrich B. and Muller N. (1997). Characterization of a cDNA-clone encoding Nc-p43, a major *Neospora caninum* tachyzoite surface protein. Parasitology 115, 581–590. 9488869 10.1017/s0031182097001650

[ref76] Hemphill A., Gajendran N., Sonda S., Fuchs N., Gottstein B., Hentrich B. and Jenkins M. (1998). Identification and characterisation of a dense granule-associated protein in *Neospora caninum* tachyzoites. International Journal for Parasitology 28, 429–438. 9559361 10.1016/s0020-7519(97)00193-8

[ref77] Hill D., Coss C., Dubey J. P., Wroblewski K., Sautter M., Hosten T., Munoz-Zanzi C., Mui E., Withers S., Boyer K., Hermes G., Coyne J., Jagdis F., Burnett A., McLeod P., Morton H., Robinson D. and McLeod R. (2011). Identification of a sporozoite-specific antigen from *Toxoplasma gondii*. Journal of Parasitology 97, 328–337. 21506817 10.1645/GE-2782.1 PMC3684278

[ref78] Hoane J. S., Yeargan M. R., Stamper S., Saville W. J., Morrow J. K., Lindsay D. S. and Howe D. K. (2005). Recombinant NhSAG1 ELISA: a sensitive and specific assay for detecting antibodies against *Neospora hughesi* in equine serum. Journal of Parasitology 91, 446–452. 15986623 10.1645/GE-395R

[ref79] Holec-Gasior L. (2013). *Toxoplasma gondii* recombinant antigens as tools for serodiagnosis of human toxoplasmosis: current status of studies. Clinical and Vaccine Immunology 20, 1343–1351. 23784855 10.1128/CVI.00117-13 PMC3889578

[ref80] Holec-Gasior L., Ferra B. and Drapala D. (2012 *a*). MIC1-MAG1-SAG1 chimeric protein, a most effective antigen for detection of human toxoplasmosis. Clinical and Vaccine Immunology 19, 1977–1979. 23035174 10.1128/CVI.00452-12 PMC3535871

[ref81] Holec-Gasior L., Ferra B., Drapala D., Lautenbach D. and Kur J. (2012 *b*). A new MIC1-MAG1 recombinant chimeric antigen can be used instead of the *Toxoplasma gondii* lysate antigen in serodiagnosis of human toxoplasmosis. Clinical and Vaccine Immunology 19, 57–63. 22116686 10.1128/CVI.05433-11 PMC3255953

[ref82] Hosseininejad M., Hosseini F., Mosharraf M., Shahbaz S., Mahzounieh M. and Schares G. (2010). Development of an indirect ELISA test using an affinity purified surface antigen (P38) for sero-diagnosis of canine *Neospora caninum* infection. Veterinary Parasitology 171, 337–342. 20434268 10.1016/j.vetpar.2010.04.003

[ref83] Howe D. K. and Sibley L. D. (1999). Comparison of the major antigens of *Neospora caninum* and *Toxoplasma gondii*. International Journal for Parasitology 29, 1489–1496. 10608434 10.1016/s0020-7519(99)00099-5

[ref84] Howe D. K., Crawford A. C., Lindsay D. and Sibley L. D. (1998). The p29 and p35 immunodominant antigens of *Neospora caninum* tachyzoites are homologous to the family of surface antigens of *Toxoplasma gondii*. Infection Immunity 66, 5322–5328. 9784539 10.1128/iai.66.11.5322-5328.1998 PMC108665

[ref85] Howe D. K., Tang K., Conrad P. A., Sverlow K., Dubey J. P. and Sibley L. D. (2002). Sensitive and specific identification of *Neospora caninum* infection of cattle based on detection of serum antibodies to recombinant Ncp29. Clinical and Diagnostic Laboratory Immunology 9, 611–615. 11986268 10.1128/CDLI.9.3.611-615.2002 PMC119992

[ref86] Huang P., Liao M., Zhang H., Lee E. G., Nishikawa Y. and Xuan X. (2007). Dense-granule protein NcGRA7, a new marker for the serodiagnosis of *Neospora caninum* infection in aborting cows. Clinical and Vaccine Immunology 14, 1640–1643. 17959821 10.1128/CVI.00251-07 PMC2168391

[ref87] Innes E. A., Lundén A., Esteban I., Marks J., Maley S., Wright S., Rae A., Harkins D., Vermeulen A., McKendrick I. J. and Buxton D. (2001). A previous infection with *Toxoplasma gondii* does not protect against a challenge with *Neospora caninum* in pregnant sheep. Parasite Immunology 23, 121–132. 11240903 10.1046/j.1365-3024.2001.00361.x

[ref88] Jacquiet P., Lienard E. and Franc M. (2010). Bovine besnoitiosis: epidemiological and clinical aspects. Veterinary Parasitology 174, 30–36. 20850933 10.1016/j.vetpar.2010.08.013

[ref89] Janitschke K., De Vos A. J. and Bigalke R. D. (1984). Serodiagnosis of bovine besnoitiosis by ELISA and immunofluorescence tests. Onderstepoort Journal of Veterinary Research 51, 239–243. 6442765

[ref90] Jenkins M. C., Wouda W. and Dubey J. P. (1997). Serological response over time to recombinant *Neospora caninum* antigens in cattle after a neosporosis-induced abortion. Clinical and Diagnostic Laboratory Immunology 4, 270–274. 9144362 10.1128/cdli.4.3.270-274.1997 PMC170517

[ref91] Jesus E. E., Almeida M. A. and Atta A. M. (2007). Anti-*Neospora*l IgG and IgE antibodies in canine neosporosis. Zoonoses and Public Health 54, 387–392. 18035978 10.1111/j.1863-2378.2007.01076.x

[ref92] Johnson A. M. and Illana S. (1991). Cloning of *Toxoplasma gondii* gene fragments encoding diagnostic antigens. Gene 99, 127–132. 2022319 10.1016/0378-1119(91)90044-c

[ref93] Johnson A. M., Illana S., McDonald P. J. and Asai T. (1989). Cloning, expression and nucleotide sequence of the gene fragment encoding an antigenic portion of the nucleoside triphosphate hydrolase of *Toxoplasma gondii*. Gene 85, 215–220. 2482826 10.1016/0378-1119(89)90484-8

[ref94] Johnson A. M., Roberts H. and Tenter A. M. (1992). Evaluation of a recombinant antigen ELISA for the diagnosis of acute toxoplasmosis and comparison with traditional antigen ELISAs. Journal of Medical Microbiology 37, 404–409. 1460660 10.1099/00222615-37-6-404

[ref95] Kasper L. H. (1987). Isolation and characterization of a monoclonal anti-P30 antibody resistant mutant of *Toxoplasma gondii*. Parasite Immunology 9, 433–445. 3627825 10.1111/j.1365-3024.1987.tb00521.x

[ref96] Kasper L. H. and Khan I. A. (1998). Antigen-specific CD8+ T cells protect against lethal toxoplasmosis in mice infected with *Neospora caninum*. Infection and Immunity 66, 1554–1560. 9529081 10.1128/iai.66.4.1554-1560.1998 PMC108088

[ref97] Kasper L. H., Bradley M. S. and Pfefferkorn E. R. (1984). Identification of stage-specific sporozoite antigens of *Toxoplasma gondii* by monoclonal antibodies. Journal of Immunology 132, 443–449. 6197455

[ref98] Kelen A. E., Ayllon-Leindl L. and Labzoffsky N. A. (1962). lndirect fluorescent antibody method in serodiagnosis of toxoplasmosis. Canadian Journal of Microbiology 8, 545–554.

[ref99] King J. S., Slapeta J., Jenkins D. J., Al-Qassab S. E., Ellis J. T. and Windsor P. A. (2010). Australian dingoes are definitive hosts of *Neospora caninum*. International Journal for Parasitology 40, 945–950. 20149793 10.1016/j.ijpara.2010.01.008

[ref100] Kobayashi T., Narabu S., Yanai Y., Hatano Y., Ito A., Imai S. and Ike K. (2013). Gene cloning and characterization of the protein encoded by the *Neospora caninum* bradyzoite-specific antigen gene BAG1. Journal of Parasitology 99, 453–458. 23245337 10.1645/12-65.1

[ref101] Kong J. T., Grigg M. E., Uyetake L., Parmley S. and Boothroyd J. C. (2003). Serotyping of *Toxoplasma gondii* infections in humans using synthetic peptides. Journal of Infectious Diseases 187, 1484–1495. 12717631 10.1086/374647

[ref102] Lally N. C., Jenkins M. C. and Dubey J. P. (1996). Evaluation of two *Neospora caninum* recombinant antigens for use in an enzyme-linked immunosorbent assay for the diagnosis of bovine neosporosis. Clinical and Diagnostic Laboratory Immunology 3, 275–279. 8705668 10.1128/cdli.3.3.275-279.1996 PMC170329

[ref103] Lally N., Jenkins M., Liddell S. and Dubey J. P. (1997). A dense granule protein (NCDG1) gene from *Neospora caninum*. Molecular and Biochemical Parasitology 87, 239–243. 9247937 10.1016/s0166-6851(97)00070-4

[ref104] Latif B. M. and Jakubek E. B. (2008). Determination of the specificities of monoclonal and polyclonal antibodies to *Neospora, Toxoplasma* and Cryptosporidium by fluorescent antibody test (FAT). Tropical Biomedicine 25, 225–231. 19287361

[ref105] Lau Y. L., Thiruvengadam G., Lee W. W. and Fong M. Y. (2011). Immunogenic characterization of the chimeric surface antigen 1 and 2 (SAG1/2) of *Toxoplasma gondii* expressed in the yeast Pichia pastoris. Parasitology Research 109, 871–878. 21455621 10.1007/s00436-011-2315-6

[ref106] Levine N. D. and Ivens V. (1981). The Coccidian Parasites (Protozoa. Apicomplexa) of Carnivores, 1st Edn. University of Illinois Press, Champaign.

[ref107] Liao M., Xuan X., Huang X., Shirafuji H., Fukumoto S., Hirata H., Suzuki H. and Fujisaki K. (2005 *a*). Identification and characterization of cross-reactive antigens from *Neospora caninum* and *Toxoplasma gondii*. Parasitology 130, 481–488. 15991490 10.1017/s0031182004006948

[ref108] Liao M., Zhang S., Xuan X., Zhang G., Huang X., Igarashi I. and Fujisaki K. (2005 *b*). Development of rapid immunochromatographic test with recombinant NcSAG1 for detection of antibodies to *Neospora caninum* in cattle. Clinical and Diagnostic Laboratory Immunology 12, 885–887. 16002641 10.1128/CDLI.12.7.885-887.2005 PMC1182214

[ref109] Liddell S., Lally N. C., Jenkins M. C. and Dubey J. P. (1998). Isolation of the cDNA encoding a dense granule associated antigen (NCDG2) of *Neospora caninum*. Molecular and Biochemical Parasitology 93, 153–158. 9662039 10.1016/s0166-6851(98)00031-0

[ref110] Lindsay D. S., Blagburn B. L. and Dubey J. P. (1990). Infection of mice with *Neospora caninum* (Protozoa: Apicomplexa) does not protect against challenge with *Toxoplasma gondii*. Infection and Immunity 58, 2699–2700. 2370115 10.1128/iai.58.8.2699-2700.1990 PMC258878

[ref111] Lindsay D. S., Lenz S. D., Dykstra C. C., Blagburn B. L. and Dubey J. P. (1998). Vaccination of mice with *Neospora caninum*: response to oral challenge with *Toxoplasma gondii* oocysts. Journal of Parasitology 84, 311–315. 9576504

[ref112] Liu J., Yu J., Wang M., Liu Q., Zhang W., Deng C. and Ding J. (2007). Serodiagnosis of *Neospora caninum* infection in cattle using a recombinant tNcSRS2 protein-based ELISA. Veterinary Parasitology 143, 358–363. 16989950 10.1016/j.vetpar.2006.08.034

[ref113] Lobato J., Silva D. A., Mineo T. W., Amaral J. D., Segundo G. R., Costa-Cruz J. M., Ferreira M. S., Borges A. S. and Mineo J. R. (2006). Detection of immunoglobulin G antibodies to *Neospora caninum* in humans: high seropositivity rates in patients who are infected by human immunodeficiency virus or have neurological disorders. Clinical and Vaccine Immunology 13, 84–89. 16426004 10.1128/CVI.13.1.84-89.2006 PMC1356624

[ref114] Lorenzi H., Khan A., Behnke M. S., Namasivayam S., Swapna L. S., Hadjithomas M., Karamycheva S., Pinney D., Brunk B. P., Ajioka J. W., Ajzenberg D., Boothroyd J. C., Boyle J. P., Darde M. L., Diaz-Miranda M. A., Dubey J. P., Fritz H. M., Gennari S. M., Gregory B. D., Kim K., Saeij J. P., Su C., White M. W., Zhu X. Q., Howe D. K., Rosenthal B. M., Grigg M. E., Parkinson J., Liu L., Kissinger J. C., Roos D. S. and David Sibley L. (2016). Local admixture of amplified and diversified secreted pathogenesis determinants shapes mosaic *Toxoplasma gondii* genomes. Nature Communications 7, 10147. 10.1038/ncomms10147 PMC4729833 26738725

[ref115] Louie K., Sverlow K. W., Barr B. C., Anderson M. L. and Conrad P. A. (1997). Cloning and characterization of two recombinant *Neospora* protein fragments and their use in serodiagnosis of bovine neosporosis. Clinical and Diagnostic Laboratory Immunology 4, 692–699. 9384291 10.1128/cdli.4.6.692-699.1997 PMC170642

[ref116] Lunde M. N. and Jacobs L. (1958). A comparison of results of hemagglutination and dye tests for toxoplasmosis in a survey of Trinidad natives. American Journal of Tropical Medicine and Hygiene 7, 523–525. 13571569 10.4269/ajtmh.1958.7.523

[ref117] Macedo A. G. Jr., Cunha J. P. Jr., Cardoso T. H., Silva M. V., Santiago F. M., Silva J. S., Pirovani C. P., Silva D. A., Mineo J. R. and Mineo T. W. (2013). SAG2A protein from *Toxoplasma gondii* interacts with both innate and adaptive immune compartments of infected hosts. Parasites & Vectors 6, 163. 23735002 10.1186/1756-3305-6-163 PMC3706231

[ref118] Maksimov P., Zerweck J., Maksimov A., Hotop A., Gross U., Pleyer U., Spekker K., Daubener W., Werdermann S., Niederstrasser O., Petri E., Mertens M., Ulrich R. G., Conraths F. J. and Schares G. (2012 *a*). Peptide microarray analysis of in silico-predicted epitopes for serological diagnosis of *Toxoplasma gondii* infection in humans. Clinical and Vaccine Immunology 19, 865–874. 22496494 10.1128/CVI.00119-12 PMC3370440

[ref119] Maksimov P., Zerweck J., Maksimov A., Hotop A., Gross U., Spekker K., Daubener W., Werdermann S., Niederstrasser O., Petri E., Mertens M., Ulrich R. G., Conraths F. J. and Schares G. (2012 *b*). Analysis of clonal type-specific antibody reactions in *Toxoplasma gondii* seropositive humans from Germany by peptide-microarray. PLoS ONE 7, e34212. 22470537 10.1371/journal.pone.0034212 PMC3314601

[ref120] Maksimov P., Zerweck J., Dubey J. P., Pantchev N., Frey C. F., Maksimov A., Reimer U., Schutkowski M., Hosseininejad M., Ziller M., Conraths F. J. and Schares G. (2013). Serotyping of *Toxoplasma gondii* in cats (*Felis domesticus*) reveals predominance of type II infections in Germany. PLoS ONE 8, e80213. 24244652 10.1371/journal.pone.0080213 PMC3820565

[ref121] Marsh A. E., Barr B. C., Packham A. E. and Conrad P. A. (1998). Description of a new *Neospora* species (Protozoa: Apicomplexa: Sarcocystidae). Journal of Parasitology 84, 983–991. 9794642

[ref122] Marsh A. E., Howe D. K., Wang G., Barr B. C., Cannon N. and Conrad P. A. (1999). Differentiation of *Neospora hughesi* from *Neospora caninum* based on their immunodominant surface antigen, SAG1 and SRS2. International Journal for Parasitology 29, 1575–1582. 10608444 10.1016/s0020-7519(99)00120-4

[ref123] McAllister M. M., Parmley S. F., Weiss L. M., Welch V. J. and McGuire A. M. (1996). An immunohistochemical method for detecting bradyzoite antigen (BAG5) in *Toxoplasma gondii*-infected tissues cross-reacts with a *Neospora caninum* bradyzoite antigen. Journal of Parasitology 82, 354–355. 8604117

[ref124] McAllister M. M., Dubey J. P., Lindsay D. S., Jolley W. R., Wills R. A. and McGuire A. M. (1998). Dogs are definitive hosts of *Neospora caninum*. International Journal for Parasitology 28, 1473–1478. 9770635

[ref125] McGarry J. W., Guy F., Trees A. J., Williams D. J. L., Davison H. C. and Björkman C. (2000). Validation and application of an inhibition ELISA to detect serum antibodies to *Neospora caninum* in different host species In: In A. Hemphill, B. Gottstein (Eds.), International Journal for Parasitology: European perspective on *Neospora caninum* pp. 880–884.

[ref126] Mineo J. R., McLeod R., Mack D., Smith J., Khan I. A., Ely K. H. and Kasper L. H. (1993). Antibodies to *Toxoplasma gondii* major surface protein (SAG-1, P30) inhibit infection of host cells and are produced in murine intestine after peroral infection. Journal of Immunology 150, 3951–3964. 7682587

[ref127] Mugridge N. B., Morrison D. A., Jakel T., Heckeroth A. R., Tenter A. M. and Johnson A. M. (2000). Effects of sequence alignment and structural domains of ribosomal DNA on phylogeny reconstruction for the protozoan family sarcocystidae. Molecular Biology and Evolution 17, 1842–1853. 11110900 10.1093/oxfordjournals.molbev.a026285

[ref128] Munday B. L. and Dubey J. P. (1986). Serological cross-reactivity between *Hammondia hammondi* and *Toxoplasma gondii* in experimentally inoculated sheep. Australian Veterinary Journal 63, 344–345. 3541885 10.1111/j.1751-0813.1986.tb02886.x

[ref129] Munday B. L. and Dubey J. P. (1988). Prevention of *Toxoplasma gondii* abortion in goats by vaccination with oocysts of *Hammondia hammondi*. Australian Veterinary Journal 65, 150–153. 3135793 10.1111/j.1751-0813.1988.tb14444.x

[ref130] Nahtman T., Jernberg A., Mahdavifar S., Zerweck J., Schutkowski M., Maeurer M. and Reilly M. (2007). Validation of peptide epitope microarray experiments and extraction of quality data. Journal of Immunological Methods 328, 1–13. 17765917 10.1016/j.jim.2007.07.015

[ref131] Neuman M. (1972). Serological survey of *Besnoitia besnoiti* (Marotel 1912) infection in Israel by immunofluorescence. Zentralblatt für Veterinarmedizin. Reihe B 19, 391–396. 10.1111/j.1439-0450.1972.tb00415.x 4559310

[ref132] Neuman de Vegvar H. E., Amara R. R., Steinman L., Utz P. J., Robinson H. L. and Robinson W. H. (2003). Microarray profiling of antibody responses against simian-human immunodeficiency virus: postchallenge convergence of reactivities independent of host histocompatibility type and vaccine regimen. Journal of Virology 77, 11125–11138. 14512560 10.1128/JVI.77.20.11125-11138.2003 PMC224970

[ref133] Nishikawa Y., Kousaka Y., Tragoolpua K., Xuan X., Makala L., Fujisaki K., Mikami T. and Nagasawa H. (2001). Characterization of *Neospora caninum* surface protein NcSRS2 based on baculovirus expression system and its application for serodiagnosis of *Neospora* infection. Journal of Clinical Microbiology 39, 3987–3991. 11682519 10.1128/JCM.39.11.3987-3991.2001 PMC88476

[ref134] Nishikawa Y., Claveria F. G., Fujisaki K. and Nagasawa H. (2002). Studies on serological cross-reaction of *Neospora caninum* with *Toxoplasma gondii* and *Hammondia heydorni*. Journal of Veterinary Medical Science 64, 161–164. 11913555 10.1292/jvms.64.161

[ref135] O'Toole D. and Jeffrey M. (1987). Congenital sporozoan encephalomyelitis in a calf. Veterinary Record 121, 563–566. 3124328

[ref136] Odberg-Ferragut C., Soete M., Engels A., Samyn B., Loyens A., Van Beeumen J., Camus D. and Dubremetz J. F. (1996). Molecular cloning of the *Toxoplasma gondii* sag4 gene encoding an 18 kDa bradyzoite specific surface protein. Molecular and Biochemical Parasitology 82, 237–244. 8946389 10.1016/0166-6851(96)02740-5

[ref137] Okeoma C. M., Williamson N. B., Pomroy W. E. and Stowell K. M. (2004). Recognition patterns of *Neospora caninum* tachyzoite antigens by bovine IgG at different IFAT titres. Parasite Immunology 26, 177–185. 15367295 10.1111/j.0141-9838.2004.00699.x

[ref138] Osawa T., Wastling J., Maley S., Buxton D. and Innes E. A. (1998). A multiple antigen ELISA to detect *Neospora*-specific antibodies in bovine sera, bovine foetal fluids, ovine and caprine sera. Veterinary Parasitology 79, 19–34. 9777723 10.1016/s0304-4017(98)00156-3

[ref139] Pare J., Hietala S. K. and Thurmond M. C. (1995 *a*). An enzyme-linked immunosorbent assay (ELISA) for serological diagnosis of *Neospora* sp. infection in cattle. Journal of Veterinary Diagnostic Investigation 7, 352–359. 7578451 10.1177/104063879500700310

[ref140] Pare J., Hietala S. K. and Thurmond M. C. (1995 *b*). Interpretation of an indirect fluorescent antibody test for diagnosis of *Neospora* sp. infection in cattle. Journal of Veterinary Diagnostic Investigation 7, 273–275. 7619917 10.1177/104063879500700222

[ref141] Parish S. M., Maag-Miller L., Besser T. E., Weidner J. P., McElwain T., Knowles D. P. and Leathers C. W. (1987). Myelitis associated with protozoal infection in newborn calves. Journal of the American Veterinary Medical Association 191, 1599–1600. 3693018

[ref142] Parmley S. F., Gross U., Sucharczuk A., Windeck T., Sgarlato G. D. and Remington J. S. (1994). Two alleles of the gene encoding surface antigen P22 in 25 strains of *Toxoplasma gondii*. Journal of Parasitology 80, 293–301. 7908967

[ref143] Parmley S. F., Weiss L. M. and Yang S. (1995). Cloning of a bradyzoite-specific gene of *Toxoplasma gondii* encoding a cytoplasmic antigen. Molecular and Biochemical Parasitology 73, 253–257. 8577335 10.1016/0166-6851(95)00100-f

[ref144] Peyron F., Lobry J. R., Musset K., Ferrandiz J., Gomez-Marin J. E., Petersen E., Meroni V., Rausher B., Mercier C., Picot S. and Cesbron-Delauw M. F. (2006). Serotyping of *Toxoplasma gondii* in chronically infected pregnant women: predominance of type II in Europe and types I and III in Colombia (South America). Microbes and Infection 8, 2333–2340. 16938480 10.1016/j.micinf.2006.03.023

[ref145] Pitel P. H., Romand S., Pronost S., Foucher N., Gargala G., Maillard K., Thulliez P., Collobert-Laugier C., Tainturier D., Fortier G. and Ballet J. J. (2003). Investigation of *Neospora* sp. antibodies in aborted mares from Normandy, France. Veterinary Parasitology 118, 1–6. 14651869 10.1016/j.vetpar.2003.10.007

[ref146] Possenti A., Cherchi S., Bertuccini L., Pozio E., Dubey J. P. and Spano F. (2010). Molecular characterisation of a novel family of cysteine-rich proteins of *Toxoplasma gondii* and ultrastructural evidence of oocyst wall localisation. International Journal for Parasitology 40, 1639–1649. 20708619 10.1016/j.ijpara.2010.06.009

[ref147] Possenti A., Fratini F., Fantozzi L., Pozio E., Dubey J. P., Ponzi M., Pizzi E. and Spano F. (2013). Global proteomic analysis of the oocyst/sporozoite of *Toxoplasma gondii* reveals commitment to a host-independent lifestyle. BMC Genomics 14, 183. 23496850 10.1186/1471-2164-14-183 PMC3616887

[ref148] Prince J. B., Auer K. L., Huskinson J., Parmley S. F., Araujo F. G. and Remington J. S. (1990). Cloning, expression, and cDNA sequence of surface antigen P22 from *Toxoplasma gondii*. Molecular and Biochemical Parasitology 43, 97–106. 2290448 10.1016/0166-6851(90)90134-8

[ref149] Reddacliff G. L., Parker S. J., Dubey J. P., Nicholls P. J., Johnson A. M. and Cooper D. W. (1993). An attempt to prevent acute toxoplasmosis in macropods by vaccination with *Hammondia hammondi*. Australian Veterinary Journal 70, 33–35. 8460987 10.1111/j.1751-0813.1993.tb00798.x

[ref150] Regidor-Cerrillo J., Garcia-Lunar P., Pastor-Fernandez I., Alvarez-Garcia G., Collantes-Fernandez E., Gomez-Bautista M. and Ortega-Mora L. M. (2015). *Neospora caninum* tachyzoite immunome study reveals differences among three biologically different isolates. Veterinary Parasitology 212, 92–99. 26324244 10.1016/j.vetpar.2015.08.020

[ref151] Reid A. J., Vermont S. J., Cotton J. A., Harris D., Hill-Cawthorne G. A., Konen-Waisman S., Latham S. M., Mourier T., Norton R., Quail M. A., Sanders M., Shanmugam D., Sohal A., Wasmuth J. D., Brunk B., Grigg M. E., Howard J. C., Parkinson J., Roos D. S., Trees A. J., Berriman M., Pain A. and Wastling J. M. (2012). Comparative genomics of the apicomplexan parasites *Toxoplasma gondii* and *Neospora caninum*: Coccidia differing in host range and transmission strategy. PLoS Pathogens 8, e1002567. 22457617 10.1371/journal.ppat.1002567 PMC3310773

[ref152] Riahi H., Darde M. L., Bouteille B., Leboutet M. J. and Pestre-Alexandre M. (1995). *Hammondia hammondi* cysts in cell cultures. Journal of Parasitology 81, 821–824. 7472890

[ref153] Riahi H., Bouteille B. and Darde M. L. (1998). Antigenic similarity between *Hammondia hammondi* and *Toxoplasma gondii* tachyzoites. Journal of Parasitology 84, 651–653. 9645881

[ref154] Riahi H., Leboutet M. J., Bouteille B., Dubremetz J. F. and Darde M. L. (1999). *Hammondia hammondi* organelle proteins are recognized by monoclonal antibodies directed against organelles of *Toxoplasma gondii*. Journal of Parasitology 85, 580–583. 10386461

[ref155] Riahi H., Leboutet M. J., Labrousse F., Bouteille B. and Darde M. L. (2000). Monoclonal antibodies to *Hammondia hammondi* allowing immunological differentiation from *Toxoplasma gondii*. Journal of Parasitology 86, 1362–1366. 11191919 10.1645/0022-3395(2000)086[1362:MATHHA]2.0.CO;2

[ref156] Risco-Castillo V., Fernandez-Garcia A. and Ortega-Mora L. M. (2004). Comparative analysis of stress agents in a simplified in vitro system of *Neospora caninum* bradyzoite production. Journal of Parasitology 90, 466–470. 15270086 10.1645/GE-3298

[ref157] Ruehlmann D., Podell M., Oglesbee M. and Dubey J. P. (1995). Canine neosporosis: a case report and literature review. Journal of the American Animal Hospital Association 31, 174–183. 7773765 10.5326/15473317-31-2-174

[ref158] Sabin A. B. (1949). Complement fixation test in toxoplasmosis and persistence of the antibody in human beings. Pediatrics 4, 443–453. 18143068

[ref159] Sabin A. B. and Feldman H. A. (1948). Dyes as microchemical indicators of a new immunity phenomenon affecting a protozoon parasite (*Toxoplasma*). Science 108, 660–663. 17744024 10.1126/science.108.2815.660

[ref160] Santana S. S., Silva D. A., Vaz L. D., Pirovani C. P., Barros G. B., Lemos E. M., Dietze R., Mineo J. R. and Cunha-Junior J. P. (2012). Analysis of IgG subclasses (IgG1 and IgG3) to recombinant SAG2A protein from *Toxoplasma gondii* in sequential serum samples from patients with toxoplasmosis. Immunology Letters 143, 193–201. 22387296 10.1016/j.imlet.2012.02.008

[ref161] Santana S. S., Gebrim L. C., Carvalho F. R., Barros H. S., Barros P. C., Pajuaba A. C., Messina V., Possenti A., Cherchi S., Reiche E. M., Navarro I. T., Garcia J. L., Pozio E., Mineo T. W., Spano F. and Mineo J. R. (2015). CCp5A protein from *Toxoplasma gondii* as a serological marker of oocyst-driven infections in humans and domestic animals. Frontiers in Microbiology 6, 1305. 26635770 10.3389/fmicb.2015.01305 PMC4656833

[ref162] Schares G., Dubremetz J. F., Dubey J. P., Barwald A., Loyens A. and Conraths F. J. (1999 *a*). *Neospora caninum*: identification of 19-, 38-, and 40-kDa surface antigens and a 33-kDa dense granule antigen using monoclonal antibodies. Experimental Parasitology 92, 109–119. 10366536 10.1006/expr.1999.4403

[ref163] Schares G., Rauser M., Zimmer K., Peters M., Wurm R., Dubey J. P., de Graaf D. C., Edelhofer R., Mertens C., Hess G. and Conraths F. J. (1999 *b*). Serological differences in *Neospora caninum*-associated epidemic and endemic abortions. Journal of Parasitology 85, 688–694. 10461950

[ref164] Schares G., Rauser M., Sondgen P., Rehberg P., Barwald A., Dubey J. P., Edelhofer R. and Conraths F. J. (2000). Use of purified tachyzoite surface antigen p38 in an ELISA to diagnose bovine neosporosis. International Journal for Parasitology 30, 1123–1130. 10996331 10.1016/s0020-7519(00)00092-8

[ref165] Schares G., Heydorn A. O., Cuppers A., Conraths F. J. and Mehlhorn H. (2001). Cyclic transmission of *Neospora caninum*: serological findings in dogs shedding oocysts. Parasitology Research 87, 873–877. 11688895 10.1007/s004360100459

[ref166] Schares G., Meyer J., Barwald A., Conraths F. J., Riebe R., Bohne W., Rohn K. and Peters M. (2003). A *Hammondia*-like parasite from the European fox (*Vulpes vulpes*) forms biologically viable tissue cysts in cell culture. International Journal for Parasitology 33, 229–234. 12670509 10.1016/s0020-7519(03)00009-2

[ref167] Schares G., Basso W., Majzoub M., Rostaher A., Scharr J. C., Langenmayer M. C., Selmair J., Dubey J. P., Cortes H. C., Conraths F. J. and Gollnick N. S. (2010). Comparative evaluation of immunofluorescent antibody and new immunoblot tests for the specific detection of antibodies against *Besnoitia besnoiti* tachyzoites and bradyzoites in bovine sera. Veterinary Parasitology 171, 32–40. 20378250 10.1016/j.vetpar.2010.03.017

[ref168] Schares G., Basso W., Majzoub M., Rostaher A., Scharr J. C., Langenmayer M. C., Selmair J., Dubey J. P., Cortes H. C., Conraths F. J., Haupt T., Purro M., Raeber A., Buholzer P. and Gollnick N. S. (2011). Evaluation of a commercial ELISA for the specific detection of antibodies against *Besnoitia besnoiti*. Veterinary Parasitology 175, 52–59. 21035269 10.1016/j.vetpar.2010.09.024

[ref169] Schares G., Langenmayer M. C., Scharr J. C., Minke L., Maksimov P., Maksimov A., Schares S., Barwald A., Basso W., Dubey J. P., Conraths F. J. and Gollnick N. S. (2013). Novel tools for the diagnosis and differentiation of acute and chronic bovine besnoitiosis. International Journal for Parasitology 43, 143–154. 23160022 10.1016/j.ijpara.2012.10.011

[ref170] Schares G., Ziller M., Herrmann D. C., Globokar M. V., Pantchev N. and Conraths F. J. (2016). Seasonality in the proportions of domestic cats shedding *Toxoplasma gondii* or *Hammondia hammondi* oocysts is associated with climatic factors. International Journal for Parasitology 46, 263–273. 26820303 10.1016/j.ijpara.2015.12.006

[ref171] Shkap V., Ungar-Waron H., Pipano E. and Greenblatt C. (1984). Enzyme linked immunosorbent assay for detection of antibodies against *Besnoitia besnoiti* in cattle. Tropical Animal Health and Production 16, 233–238. 6441325 10.1007/BF02265330

[ref172] Shkap V., Reske A., Pipano E., Fish L. and Baszler T. (2002). Immunological relationship between *Neospora caninum* and *Besnoitia besnoiti*. Veterinary Parasitology 106, 35–43. 11992709 10.1016/s0304-4017(02)00030-4

[ref173] Sibley L. D., Pfefferkorn E. R. and Boothroyd J. C. (1991). Proposal for a uniform genetic nomenclature in *Toxoplasma gondii*. Parasitology Today 7, 327–328. 15463406 10.1016/0169-4758(91)90210-f

[ref174] Silva D. A., Lobato J., Mineo T. W. and Mineo J. R. (2007). Evaluation of serological tests for the diagnosis of *Neospora caninum* infection in dogs: optimization of cut off titers and inhibition studies of cross-reactivity with *Toxoplasma gondii*. Veterinary Parasitology 143, 234–244. 16973287 10.1016/j.vetpar.2006.08.028

[ref175] Slapeta J. R., Koudela B., Votypka J., Modry D., Horejs R. and Lukes J. (2002). Coprodiagnosis of *Hammondia heydorni* in dogs by PCR based amplification of ITS 1 rRNA: differentiation from morphologically indistinguishable oocysts of *Neospora caninum*. Veterinary Journal 163, 147–154. 10.1053/tvjl.2001.0599 12093189

[ref176] Soares R. M., Cortez L. R., Gennari S. M., Sercundes M. K., Keid L. B. and Pena H. F. (2009). Crab-eating fox (*Cerdocyon thous*), a South American canid, as a definitive host for *Hammondia heydorni*. Veterinary Parasitology 162, 46–50. 19303215 10.1016/j.vetpar.2009.02.003

[ref177] Sohn C. S., Cheng T. T., Drummond M. L., Peng E. D., Vermont S. J., Xia D., Cheng S. J., Wastling J. M. and Bradley P. J. (2011). Identification of novel proteins in *Neospora caninum* using an organelle purification and monoclonal antibody approach. PLoS ONE 6, e18383. 21483743 10.1371/journal.pone.0018383 PMC3070720

[ref178] Sonda S., Fuchs N., Connolly B., Fernandez P., Gottstein B. and Hemphill A. (1998). The major 36 kDa *Neospora caninum* tachyzoite surface protein is closely related to the major *Toxoplasma gondii* surface antigen. Molecular and Biochemical Parasitology 97, 97–108. 9879890 10.1016/s0166-6851(98)00133-9

[ref179] Sonda S., Fuchs N., Gottstein B. and Hemphill A. (2000). Molecular characterization of a novel microneme antigen in *Neospora caninum*. Molecular and Biochemical Parasitology 108, 39–51. 10802317 10.1016/s0166-6851(00)00200-0

[ref180] Sondgen P., Peters M., Barwald A., Wurm R., Holling F., Conraths F. J. and Schares G. (2001). Bovine neosporosis: immunoblot improves foetal serology. Veterinary Parasitology 102, 279–290. 11731071 10.1016/s0304-4017(01)00543-x

[ref181] Sousa S., Ajzenberg D., Vilanova M., Costa J. and Darde M. L. (2008). Use of GRA6-derived synthetic polymorphic peptides in an immunoenzymatic assay to serotype *Toxoplasma gondii* in human serum samples collected from three continents. Clinical and Vaccine Immunology 15, 1380–1386. 18667636 10.1128/CVI.00186-08 PMC2546681

[ref182] Sousa S., Ajzenberg D., Marle M., Aubert D., Villena I., da Costa J. C. and Darde M. L. (2009). Selection of polymorphic peptides from GRA6 and GRA7 sequences of *Toxoplasma gondii* strains to be used in serotyping. Clinical and Vaccine Immunology 16, 1158–1169. 19494084 10.1128/CVI.00092-09 PMC2725539

[ref183] Srinivasan S., Baszler T., Vonlaufen N., Leepin A., Sanderson S. J., Wastling J. M. and Hemphill A. (2006). Monoclonal antibody directed against *Neospora caninum* tachyzoite carbohydrate epitope reacts specifically with apical complex-associated sialylated beta tubulin. Journal of Parasitology 92, 1235–1243. 17304800 10.1645/GE-889R.1

[ref184] Staubli D., Nunez S., Sager H., Schares G. and Gottstein B. (2006). *Neospora caninum* immunoblotting improves serodiagnosis of bovine neosporosis. Parasitology Research 99, 648–658. 16718512 10.1007/s00436-006-0207-y

[ref185] Stenlund S., Björkman C., Holmdahl O. J., Kindahl H. and Uggla A. (1997). Characterization of a Swedish bovine isolate of *Neospora caninum*. Parasitology Research 83, 214–219. 9089715 10.1007/s004360050236

[ref186] Sundermann C. A., Estridge B. H., Branton M. S., Bridgman C. R. and Lindsay D. S. (1997). Immunohistochemical diagnosis of *Toxoplasma gondii*: potential for cross- reactivity with *Neospora caninum*. Journal of Parasitology 83, 440–443. 9194824

[ref187] Tenter A. M., Vietmeyer C. and Johnson A. M. (1992). Development of ELISAs based on recombinant antigens for the detection of *Toxoplasma gondii*-specific antibodies in sheep and cats. Veterinary Parasitology 43, 189–201. 1413451 10.1016/0304-4017(92)90160-b

[ref188] Tenter A. M., Heckeroth A. R. and Weiss L. M. (2000). *Toxoplasma gondii*: from animals to humans. International Journal for Parasitology 30, 1217–1258. 11113252 10.1016/s0020-7519(00)00124-7 PMC3109627

[ref190] Tunev S. S., McAlilster M. M., Anderson-Sprecher R. C. and Weiss L. M. (2002). *Neospora caninum in vitro*: evidence that the destiny of a parasitophorous vacuole depends on the phenotype of the progenitor zoite. Journal of Parasitology 88, 1095–1099. 12537100 10.1645/0022-3395(2002)088[1095:NCIVET]2.0.CO;2 PMC3109616

[ref191] Uchida Y., Ike K., Kurotaki T., Ito A. and Imai S. (2004). Monoclonal antibodies preventing invasion of *Neospora caninum* tachyzoites into host cells. Journal of Veterinary Medical Science 66, 1355–1358. 15585948 10.1292/jvms.66.1355

[ref192] Uzêda R. S., Schares G., Ortega-Mora L. M., Madruga C. R., Aguado-Martinez A., Corbellini L. G., Driemeier D. and Gondim L. F. (2013). Combination of monoclonal antibodies improves immunohistochemical diagnosis of *Neospora caninum*. Veterinary Parasitology 197, 477–486. 23927916 10.1016/j.vetpar.2013.07.008

[ref193] Veronesi F., Diaferia M., Mandara M. T., Marenzoni M. L., Cittadini F. and Piergili Fioretti D. (2008). *Neospora* spp. infection associated with equine abortion and/or stillbirth rate. Veterinary Research Communications 32(Suppl 1), S223–S226. 18696243 10.1007/s11259-008-9155-6

[ref194] Vonlaufen N., Guetg N., Naguleswaran A., Muller N., Björkman C., Schares G., von Blumroeder D., Ellis J. and Hemphill A. (2004). *In vitro* induction of *Neospora caninum* bradyzoites in vero cells reveals differential antigen expression, localization, and host-cell recognition of tachyzoites and bradyzoites. Infection and Immunity 72, 576–583. 14688139 10.1128/IAI.72.1.576-583.2004 PMC343979

[ref195] Walls K. W., Bullock S. L. and English D. K. (1977). Use of the enzyme-linked immunosorbent assay (ELISA) and its microadaptation for the serodiagnosis of toxoplasmosis. Journal of Clinical Microbiology 5, 273–277. 323276 10.1128/jcm.5.3.273-277.1977 PMC274581

[ref196] Walsh C. P., Vemulapalli R., Sriranganathan N., Zajac A. M., Jenkins M. C. and Lindsay D. S. (2001). Molecular comparison of the dense granule proteins GRA6 and GRA7 of *Neospora hughesi* and *Neospora caninum*. International Journal for Parasitology 31, 253–258. 11226451 10.1016/s0020-7519(00)00169-7

[ref197] Walzer K. A., Adomako-Ankomah Y., Dam R. A., Herrmann D. C., Schares G., Dubey J. P. and Boyle J. P. (2013). *Hammondia hammondi*, an avirulent relative of *Toxoplasma gondii*, has functional orthologs of known *T. gondii* virulence genes. Proceedings of the National Academy of Sciences of the United States of America 110, 7446–7451. 23589877 10.1073/pnas.1304322110 PMC3645575

[ref198] Walzer K. A., Wier G. M., Dam R. A., Srinivasan A. R., Borges A. L., English E. D., Herrmann D. C., Schares G., Dubey J. P. and Boyle J. P. (2014). *Hammondia hammondi* harbors functional orthologs of the host-modulating effectors GRA15 and ROP16 but is distinguished from *Toxoplasma gondii* by a unique transcriptional profile. Eukaryotic Cell 13, 1507–1518. 25280815 10.1128/EC.00215-14 PMC4248688

[ref199] Wasmuth J. D., Pszenny V., Haile S., Jansen E. M., Gast A. T., Sher A., Boyle J. P., Boulanger M. J., Parkinson J. and Grigg M. E. (2012). Integrated bioinformatic and targeted deletion analyses of the SRS gene superfamily identify SRS29C as a negative regulator of *Toxoplasma* virulence. MBio 3. doi: 10.1128/mBio.00321-12. PMC3509429 23149485

[ref200] Weiland G., Rommel M. and von Seyerl F. (1979). Serological cross-reactions between *Toxoplasma* and hammondia. Zentralbl Bakteriol Orig A 244, 391–393. 388947

[ref201] Weiss L. M., LaPlace D., Tanowitz H. B. and Wittner M. (1992). Identification of *Toxoplasma gondii* bradyzoite-specific monoclonal antibodies. Journal of Infectious Diseases 166, 213–215. 1376757 10.1093/infdis/166.1.213

[ref202] Weiss L. M., Ma Y. F., Halonen S., McAllister M. M. and Zhang Y. W. (1999). The *in vitro* development of *Neospora caninum* bradyzoites. International Journal for Parasitology 29, 1713–1723. 10608459 10.1016/s0020-7519(99)00130-7 PMC3086365

[ref203] Williams D. J., McGarry J., Guy F., Barber J. and Trees A. J. (1997). Novel ELISA for detection of *Neospora*-specific antibodies in cattle. Veterinary Record 140, 328–331. 9106971 10.1136/vr.140.13.328

[ref204] Wouda W., Brinkhof J., van Maanen C., de Gee A. L. and Moen A. R. (1998). Serodiagnosis of neosporosis in individual cows and dairy herds: a comparative study of three enzyme-linked immunosorbent assays. Clinical and Diagnostic Laboratory Immunology 5, 711–716. 9729540 10.1128/cdli.5.5.711-716.1998 PMC95644

[ref205] Wyrosdick H. M. and Schaefer J. J. (2015). *Toxoplasma gondii*: history and diagnostic test development. Animal Health Research Reviews 16, 150–162. 26568360 10.1017/S1466252315000183

[ref206] Yang C. D., Chang G. N. and Chao D. (2004). Protective immunity against *Toxoplasma gondii* in mice induced by a chimeric protein rSAG1/2. Parasitology Research 92, 58–64. 14605877 10.1007/s00436-003-0992-5

[ref207] Ybanez R. H., Terkawi M. A., Kameyama K., Xuan X. and Nishikawa Y. (2013). Identification of a highly antigenic region of subtilisin-like serine protease 1 for serodiagnosis of *Neospora caninum* infection. Clinical and Vaccine Immunology 20, 1617–1622. 23966554 10.1128/CVI.00352-13 PMC3807189

[ref208] Yin J., Qu G., Cao L., Li Q., Fetterer R., Feng X., Liu Q., Wang G., Qi D., Zhang X., Miramontes E., Jenkins M., Zhang N. and Tuo W. (2012). Characterization of *Neospora caninum* microneme protein 10 (NcMIC10) and its potential use as a diagnostic marker for neosporosis. Veterinary Parasitology 187, 28–35. 22284302 10.1016/j.vetpar.2012.01.003

[ref209] Zhang H., Compaore M. K., Lee E. G., Liao M., Zhang G., Sugimoto C., Fujisaki K., Nishikawa Y. and Xuan X. (2007 *a*). Apical membrane antigen 1 is a cross-reactive antigen between *Neospora caninum* and *Toxoplasma gondii*, and the anti-NcAMA1 antibody inhibits host cell invasion by both parasites. Molecular and Biochemical Parasitology 151, 205–212. 17156863 10.1016/j.molbiopara.2006.11.005

[ref210] Zhang H., Lee E. G., Liao M., Compaore M. K., Zhang G., Kawase O., Fujisaki K., Sugimoto C., Nishikawa Y. and Xuan X. (2007 *b*). Identification of ribosomal phosphoprotein P0 of *Neospora caninum* as a potential common vaccine candidate for the control of both neosporosis and toxoplasmosis. Molecular and Biochemical Parasitology 153, 141–148. 17412435 10.1016/j.molbiopara.2007.02.012

[ref211] Zhang H., Lee E. G., Yu L., Kawano S., Huang P., Liao M., Kawase O., Zhang G., Zhou J., Fujisaki K., Nishikawa Y. and Xuan X. (2011). Identification of the cross-reactive and species-specific antigens between *Neospora caninum* and *Toxoplasma gondii* tachyzoites by a proteomics approach. Parasitology Research 109, 899–911. 21461729 10.1007/s00436-011-2332-5

